# Protease‐Mediated Synthesis of Zein Nanofibrils: From Structural Elucidation to Functional Application

**DOI:** 10.1002/advs.202414606

**Published:** 2025-03-31

**Authors:** Mingqin Li, Tonghui Jin, Simone Wüthrich, Jiangtao Zhou, Qiyao Sun, Ting Li, Zhou Dong, Eva Maria Zunzuneigui Bru, Raffaele Mezzenga

**Affiliations:** ^1^ Department of Health Sciences and Technology ETH Zurich Schmelzbergstrasse 9 Zurich 8092 Switzerland; ^2^ Functional Genomics Center Zurich UZH/ETH Zurich Winterthurestrasse 190 Zurich 8057 Switzerland; ^3^ School of Food Science and Technology Jiangnan University Lihu Road 1800 Wuxi 214122 China; ^4^ Department of Materials ETH Zurich Wolfgang‐Pauli‐Strasse 10 Zurich 8093 Switzerland

**Keywords:** aqueous environment, enzymatic hydrolysis, functional building blocks, zein nanofibrils

## Abstract

The fibrillization of plant‐based proteins enhances their functionality, enabling potential applications in food and sustainable materials. Zein, a highly hydrophobic protein from corn, is a versatile industrial ingredient, but its functionality is limited to environments containing high levels of organic solvents. This study aims to develop a protease‐assisted approach for synthesizing zein nanofibrils as functional building blocks, eliminating the need for organic solvents in the conventional process. Through proteomics, microscopy, and spectroscopy, the bioprocess and structural features of these novel nanofibrils are characterized. The results reveal that over 50% of α‐zein sequence is prone to fibrillization, with pepsin demonstrating a clear advantage in efficiently releasing fibrillization‐prone peptide segments (bioconversion > 70%) and producing a peptide mixture suitable for self‐assembly. The fibrillization process is significantly enhanced by increasing peptide concentration and adding the anionic surfactant sodium dodecyl sulfate, which can lead to the formation of semiflexible fibrils with amyloid‐like β‐sheet structures. These nanofibrils outperformed native zein as emulsifiers in high internal phase emulsions and are able to form fibrous hydrogels. The protease‐assisted fibrillization process achieved in this study provides an effective solution for expanding applications of zein or corn proteins in a purely aqueous environment.

## Graphical abstract

The globular shape and hydrophobicity of zein made it unsuitable to be processed in pure aqueous environment. Pepsin‐mediated hydrolysis converts zein into water‐soluble and fibril‐forming peptides with a bioconversion rate exceeding 70%, enabling the self‐assembly of amyloid‐like nanofibrils. These nanofibrils are promising natural emulsifier and gelling reagent.

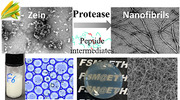

## 1 Introduction

Plant‐based proteins are gaining momentum as viable alternatives to animal sources or synthetic polymers, due to their renewability, natural abundance, and biocompatibility.^[^ ^1^ ^]^ Fibrillization is an effective approach to functionalize plant proteins, which converts globular protein into nanofibril rich in β‐sheet offering superior self‐assembly and mechanical properties as building blocks.^[^ ^2^ ^]^ Sharing the same structural hallmark of cross‐β strands with the pathological and functional amyloids formed in vivo, these nanofibrils are synthesized in vitro under specific conditions (pH, temperature, agitation, solvent polarity, and presence of metal ions and/or surfactants), in which proteins undergo unfolding and eventually hydrolysis, exposing fibrillization‐prone segments and finally reorganizing into nanofibrils with cross‐β strands structure.^[^ ^3^, ^4^ ^]^

Although fibrillization‐prone segments are widely found in protein sequences,^[^ ^5^ ^]^ achieving fibrillization has been challenging for many plant‐based proteins due to their diverse compositions, complex quaternary structure, and limited solubility. Proteases, enzymes that cleave proteins at specific amino acid residues and induce protein unfolding or hydrolysis, have been used to facilitate the fibrillization of plant proteins with complex and bulky structures. For example, wheat gluten, particularly the gliadin fraction, cannot be fibrillized by simple temperature and pH adjustments. Long‐time enzymatic hydrolysis, ranging from 38 h to two weeks, by trypsin or protease K facilitated fibril formation via releasing soluble fibrillization‐prone segments from wheat gluten.^[^ ^6^, ^7^ ^]^ Enzymatic hydrolysis was also employed to modulate the composition, morphology, and nanofibrils functionality from other food proteins, such as soy protein isolates.^[^ ^8^ ^]^ Although protease‐assisted approach is generally perceived with increased cost, it is a safe, non‐toxic, and energy‐efficient means to facilitate the reassembly of plant proteins into innovative mesoscopic and macroscopic structures, which expands their applications from nutraceutical and future foods to regenerative tissue scaffolds and functional materials.^[^ ^9^, ^10^, ^11^, ^12^ ^]^

Corn, the third most‐produced global staple crop after wheat and rice, is a valuable source of plant‐based proteins. Zein, as the major storage protein of corn endosperm, constitutes 60–80% of corn proteins and is commercially extracted from corn gluten meal, a by‐product of corn starch and oil production.^[^ ^13^, ^14^ ^]^ Despite its limitation for food applications due to low solubility and deficiencies in essential amino acids, such as lysine and tryptophan, zein has broad industrial applications, including thermoplastics, textiles, adhesives, and coatings, primarily attributable to its self‐assembling properties.^[^ ^15^ ^]^ Along with wheat gliadin and barley hordein, zein belongs to the prolamin category. The sequence of zein is composed of a high proportion of hydrophobic amino acids (≈70%, e.g., proline, phenylalanine, leucine, and alanine) and contains multiple repeating glutamine regions (QQQQ_(n)_), which leads to rich helical structures in its native form.^[^ ^16^, ^17^ ^]^ Previous studies have demonstrated that zein nanofibrils form in alcohol–water binary solvent (60–75% of alcohol) at pH 4–9.5, while nanospheres or irregular aggregates were observed outside of this pH range and solvent polarity.^[^ ^18^, ^19^ ^]^ Zein nanofibrils exhibit a “beaded” morphology, in which nanospheres form initially via H‐bonding and nucleation, eventually joining into a chain. This process involves a structural transition from α‐helix to β‐sheet.^[^ ^19^ ^]^ A considerable amount of non‐polar solvent is required for zein to counterbalance its strong hydrophobic interactions. Additionally, research suggests that disrupting zein's native structure enhances its ability to form β‐sheet‐rich materials, which improves its elasticity and makes it suitable for various applications.^[^ ^17^ ^]^

**Scheme 1:**
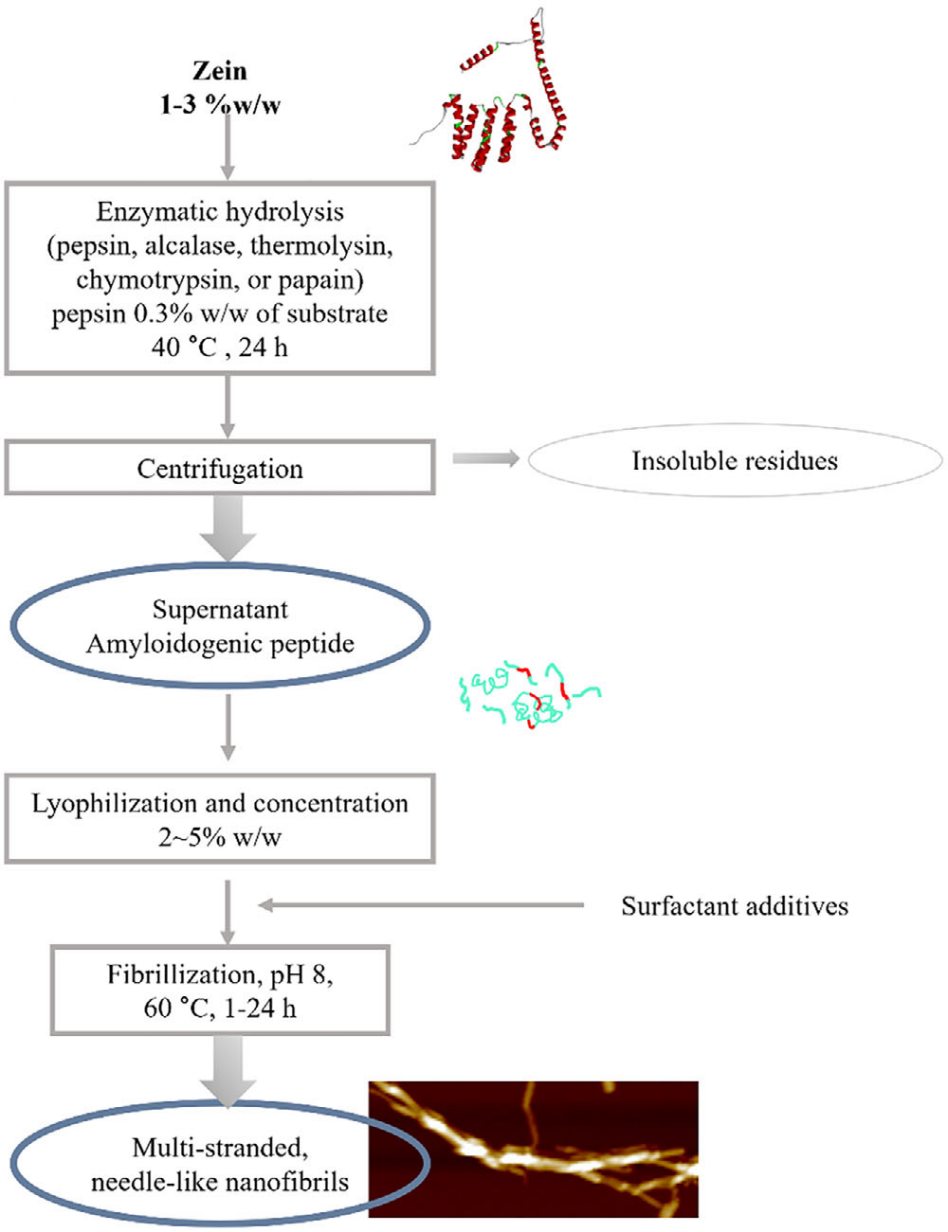
Bioprocess flowchart.

To develop a viable alternative to the approaches mentioned above, we hypothesize that enzymatic hydrolysis can provide a distinct route to facilitate zein fibrillization by modulating its hydrophobic–hydrophilic balance and exposing its fibrillization‐prone regions. To the best of the authors’ knowledge, the combination of enzymatic hydrolysis with zein fibrillization has not yet been previously explored. This study aims to synthesize zein nanofibrils through a protease‐assisted bioprocess in an aqueous environment, and to elucidate the structure and functionality of these nanofibrils. The protease‐assisted process for fibrillization is depicted as **Scheme** **1**. To use the enzyme efficiently, the bioconversion yield of fibrillization‐prone peptides over time and their mass profile from five different proteases (pepsin, alcalase, thermolysin, chymotrypsin, and papain) were compared. Among them, pepsin‐derived hydrolysate showed the greatest potential for fibrillization. The fibrillization kinetics of zein peptide was revealed by Thioflavin T (ThT) assay. The structure and morphology of zein nanofibrils were thoroughly characterized from molecular to mesoscopic levels using microscopy (TEM, AFM), spectroscopy (FTIR, CD), and proteomic analysis. Furthermore, we demonstrated the ability of these nanofibrils to stabilize oil–water interfaces and to form fibrous gels, thus establishing the foundation for the use of the resultant zein nanofibrils as sustainable building blocks for a multitude of applications.

## 2 Results and Discussion

### 2.1 Enzymatic Hydrolysis of Zein

**Figure 1:**
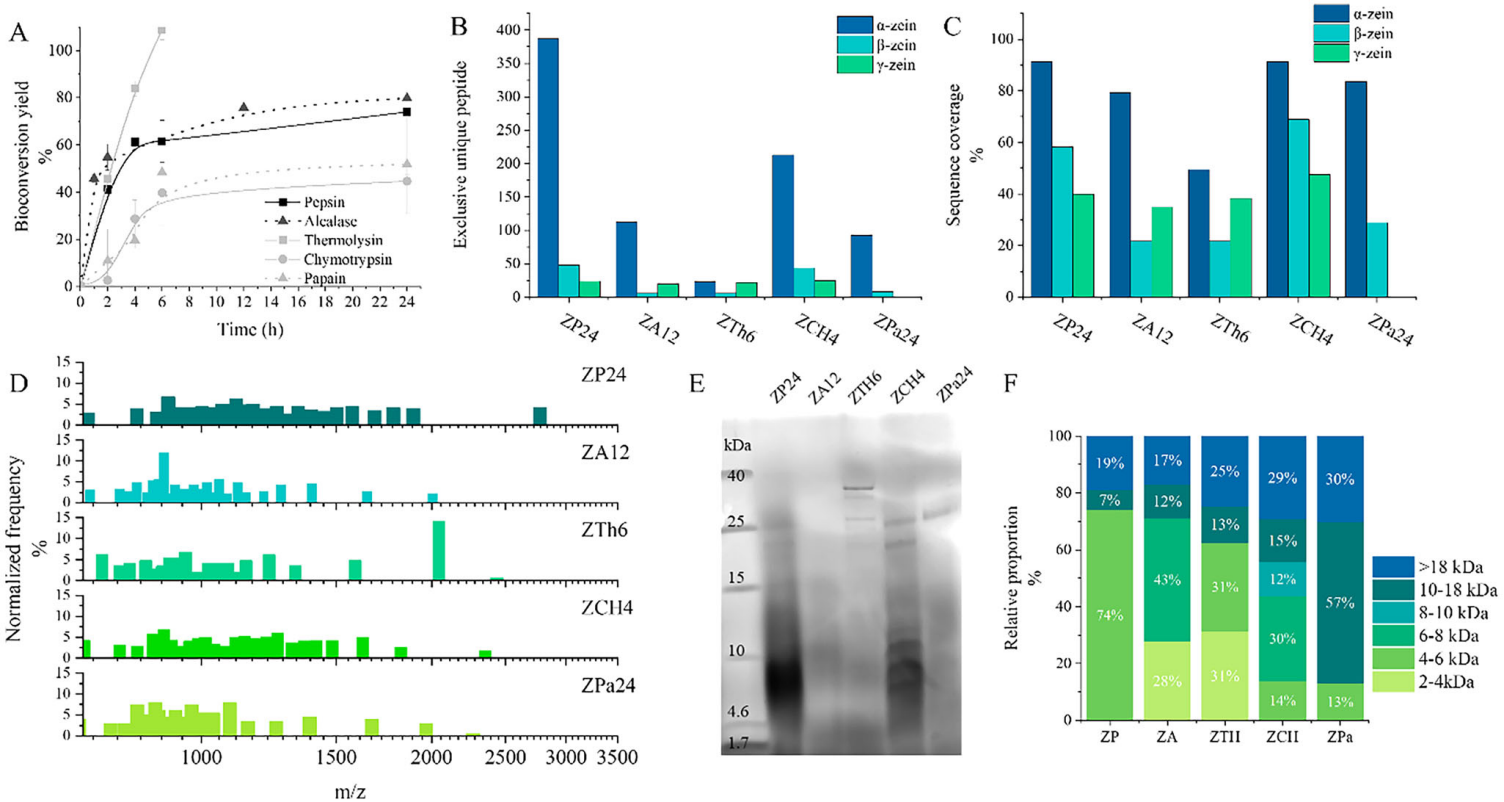
Protease‐assisted hydrolysis of zein. A) bioconversion yield of zein into hydrolysate peptide mixture by different proteases over time; B) exclusive unique peptides detected from each hydrolysate by LC‐MS/MS and their origin from different zein fractions; C) percentage of protein sequence that has been covered by the detected peptide fragments in the hydrolysate; D) product profile of peptide mixtures obtained from different proteases between 600–3500 Da, characterized  by mass spectra of their 1H^+^ isotopic mass‐to‐charge ratio(D) and E) & F) product profile between 2000–10000 Da characterized by tricine SDS‐PAGE.

In an aqueous environment, the heat treatment of zein under both acidic and alkaline conditions with shear resulted in agglomeration, while protease treatment promoted the solubilization of zein (Figure S1, Supporting Information). All five selected proteases hydrolyzed zein, varying in the efficiency and the molecular weight profile of their hydrolysate peptide mixtures. The bioconversion yields of insoluble zein to soluble hydrolysates over 24 h are shown in **Figure** **1**A. Hydrolysis catalyzed by thermolysin was the most efficient, achieving complete dissolution of zein within 6 h. In contrast, hydrolysis by papain and chymotrypsin resulted in less than 50% bioconversion after 24 h. Pepsin and alcalase achieved bioconversions of 74.0% and 79.6%, respectively, after 24 h of hydrolysis. The molecular weight profile of the zein and its hydrolysates were monitored via Tricine SDS‐PAGE (Figure S2, Supporting Information), revealing similar electrophoretic patterns for peptides generated by pepsin, thermolysin, and papain. However, long hydrolysis times with alcalase and chymotrypsin (24 h) led to fading on bands seen in short hydrolysis times (<12 h), likely due to continuous cleavage of the peptide products. Hydrolysates collected at 24, 12, 6, 4, and 24 h from hydrolysis by pepsin (ZP24), alcalase (ZA12), thermolysin (ZTh6), chymotrypsin (ZCH4), and papain (ZPa24), respectively, were further analyzed. The protein content of these hydrolysates was determined to be > 95% (dry weight) based on the total nitrogen measurement.

Zein can be divided into four fractions by molecular weight and structure: α, β, γ, and δ zein. Commercial zein is predominantly α‐zein (80–85%) with two subunits at a molecular weight of 20 and 24 kDa. Other fractions‐β (14 kDa), γ (two units, 27 and 18 kDa), and δ (one unit between 10–18 kDa) are minor components, accounting for ≈10%, 10–15%, and 1–5% of zein, respectively.^[^ ^16^ ^]^ Proteomic analysis indicated the presence of peptide derived from α, β, and γ‐zein in hydrolysate samples, except that from papain, which contained peptides only from α and β‐zein (Figure 1B,C). Exclusive unique peptides, matched by a minimum of eight amino acids, are used to indicate the origin of the peptide in the hydrolysate.^[^ ^20^ ^]^ The highest counts of exclusive unique peptides were from α‐zein (Figure 1B). The analysis also revealed a relatively high sequence coverage of α‐zein compared to other zein fractions (Figure 1C), implying that α‐zein is very prone to disintegration into peptide by selected proteases. Among the enzymes, pepsin produced the highest counts of exclusive unique peptides from α‐zein and achieved over 90% of sequence coverage of α‐zein. The mass spectra of the peptide mixtures exhibited a distribution predominantly between 600–3000 Da (Figure 1D). Specifically, ZP24 and ZCH4 had relatively balanced distributions between 900 and 2000 Da; whereas ZA12, ZPa24, and ZTh6 contained smaller peptide fragments, concentrated ≈1000 Da. Larger peptide fragments (2–10 kDa) in these hydrolysates were further characterized based on electrophoretic pattern (Figure 1E,F). ZP24 and ZTh6 contained over 60% of these fragments between 2–6 kDa, while the other samples (ZA12, ZPa24, ZCH4) primarily consisted of larger fragments ranging from 6 to18 kDa (50–60%).

Previous studies have used enzymatic hydrolysis to fractionate bioactive peptides from corn proteins, which exhibit antioxidant, neuroprotective properties, typically with molecular weights lower than 1500 Da.^[^ ^13^, ^21^ ^]^ To date, no zein‐derived peptides prone to fibrillization have been reported. However, fibrillization‐prone peptides have been identified in nanofibrils from other plant proteins, including potato, soy, and rice proteins, with molecular weights ranging from 800 to 3000 Da, and consisting of multiple populations rather than single pure forms.^[^ ^22^, ^23^, ^24^ ^]^ The current study observed that enzymatic hydrolysis significantly improved zein dispersibility in aqueous solution and efficiently released abundant peptide segments from α‐zein into the solution. Peptide mixtures of different enzymatic hydrolysis were further investigated to elucidate their assembling behavior.

### 2.2 Effect of Enzymatic Hydrolysis on Physicochemical‐Properties‐Related Assembly Behaviors

**Figure 2:**
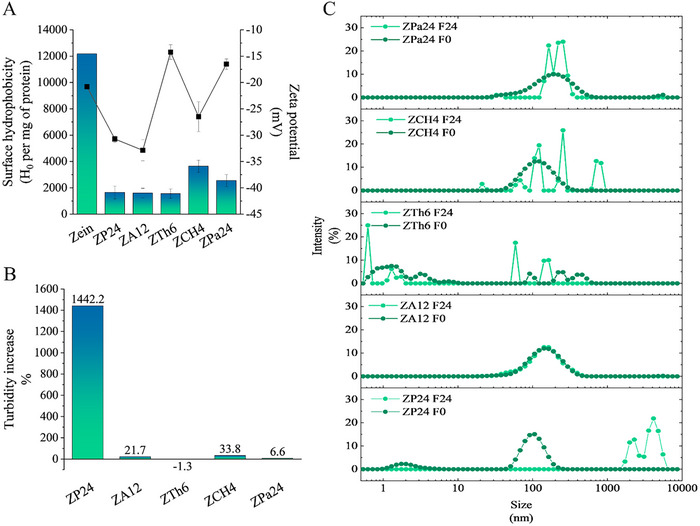
Physicochemical properties of protein materials derived from zein. A) surface hydrophobicity and zeta potential; B) aggregation propensity as indicated by increased turbidity; C) aggregation propensity as indicated by changes in hydrodynamic size. F0 and F24 refer to heat treatment for 0 h and 24 h, respectively.

Surface hydrophobicity, reflecting the number and size of hydrophobic cleft on protein surface, and propensity to form aggregates via hydrophobic interactions, was quantified by measuring the fluorescence intensity of ANS upon binding to the protein materials.^[^ ^25^ ^]^ All enzymatic hydrolysates showed significantly lower surface hydrophobicity than native zein in aqueous solution at pH 8 (**Figure** **2**A), indicating that hydrophilic functional groups, initially buried in zein, were exposed. This result is consistent with previous research that reported a continuous decrease in zein surface hydrophobicity during protease treatments as a function of time.^[^ ^26^, ^27^ ^]^ Enzymatic hydrolysis also altered the surface charge of the protein molecules, as reflected by their zeta potential (Figure 2A). Compared to native zein, ZP24, ZA12, and ZCH4 showed more negative charge at pH 8, indicating stronger electrostatic repulsion, whereas, ZTh6 and ZPa24 exhibited weaker electrostatic repulsion. Negative charges are known to enhance the protein–water interaction and stabilize protein colloidal systems, thus promoting zein assembly during heat treatment or solvent evaporation.^[^ ^18^, ^28^ ^]^

The aggregation propensity induced by heat treatment was investigated through turbidity and particle size measurements. Changes in solution turbidity are related to the presence of micron‐level spherical or rod‐like aggregates.^[^ ^29^ ^]^ Samples were subjected to heat treatment at 60 °C, pH 8 for 24 h under constant shear. A significant increase in turbidity was observed after the pepsin treatment (ZP24) (Figure 2B), which corresponded to an increase in hydrodynamic size from 112.7 at 0 h to 4123.7 nm at 24 h (Figure 2C). No apparent increase in turbidity or hydrodynamic size toward micron‐scale was observed in other peptide mixtures. These results suggest that ZP24 has a higher aggregation propensity than other zein hydrolysates. Excessive hydrophobic interactions and Van der Waals interactions can lead to irregular aggregation and coagulation of zein in aqueous environments, obstructing its structural rearrangement.^[^ ^28^ ^]^ Enzymatic hydrolysis, particularly with pepsin, overcomes this limitation and generates a hydrolysate peptide mixture (ZP24) with a balance between hydrophobic interaction and electrostatic repulsion, which favors the formation of micron‐level self‐assemblies.

It is worth noting that prolamin proteins from wheat gluten and corn tend to self‐assemble into stable structures under neutral to basic conditions during heat‐induced processes, which is different from the requisite acidic conditions for fibrillization of the globulin‐type of food protein, such as β‐lactoglobulin, seed, and pseudocereal proteins.^[^ ^30^, ^31^, ^32^, ^33^ ^]^ This behavior can be ascribed to the high glutamine content in prolamins, which undergoes deamidation in an acidic condition over 60 °C. The reaction reduces the polarity of the molecules, thereby disrupting molecular interactions necessary for β‐sheet fibrillar assembly.^[^ ^28^ ^]^ Therefore, the physicochemical changes and aggregation behaviors of peptide mixtures were studied at pH 8 to obtain clear evidence for further investigation on their fibrillization.

### 2.3 Fibrillization Kinetics and Fibril Morphology

**Figure 3:**
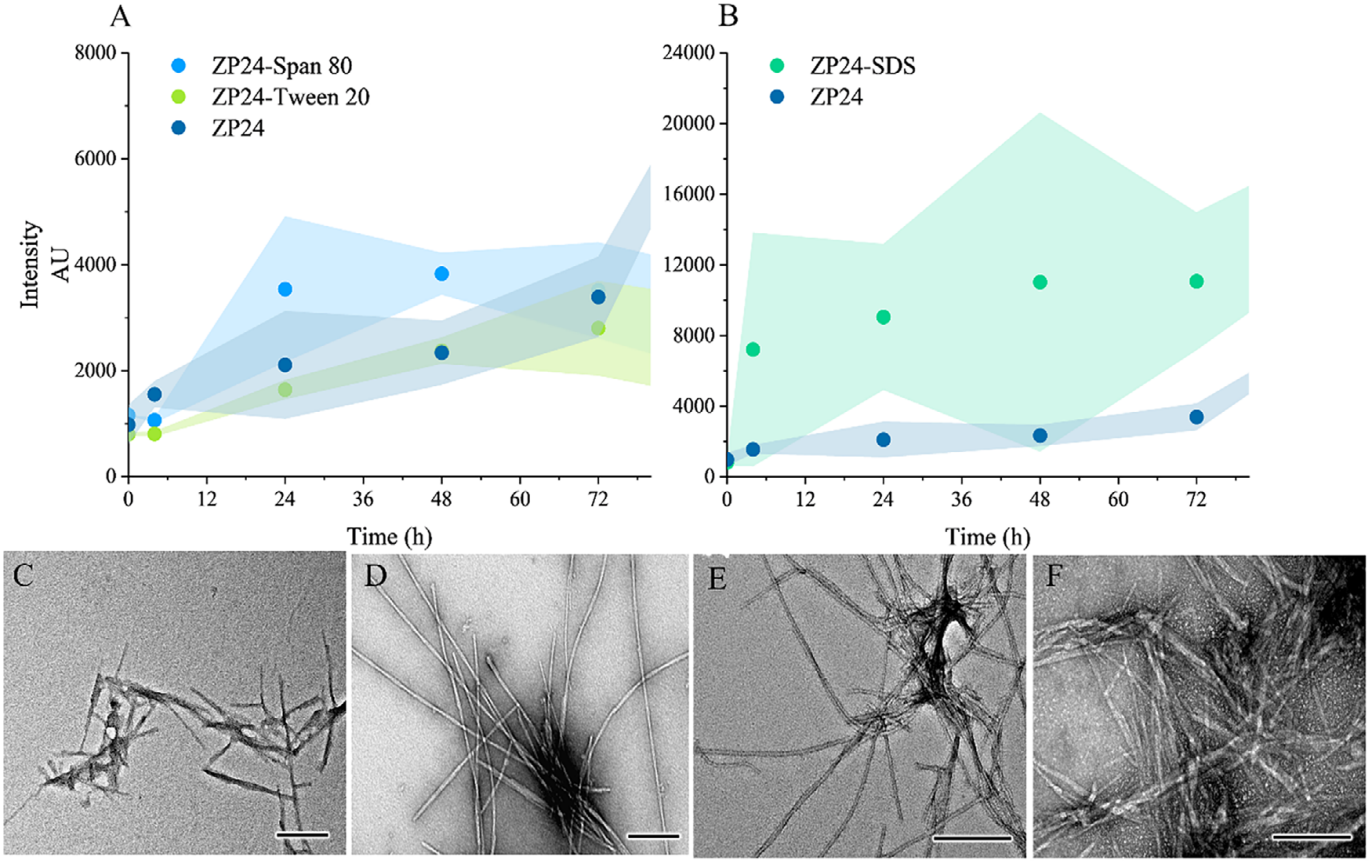
Fibrillization of ZP24 alone and in the presence of different surfactants as additives. Fibrillization process monitored by ThT assay (A and B); TEM images of nanofibrils from the fibrillization of ZP24 alone, 72 h (C), ZP24 ‐SDS, 4 h (D), ZP24‐Span 80, 24 h (E), ZP24‐Tween 20, 72 h (F). Scale bar represents 200 nm. These nanofibrils were taken from samples at the time points where the ThT signal approached saturation. The fibrillization was carried out at 2 wt.%  of ZP24, at pH 8, 60 °C. The shadow along the plots indicates the standard deviation of the data.

**Figure 4:**
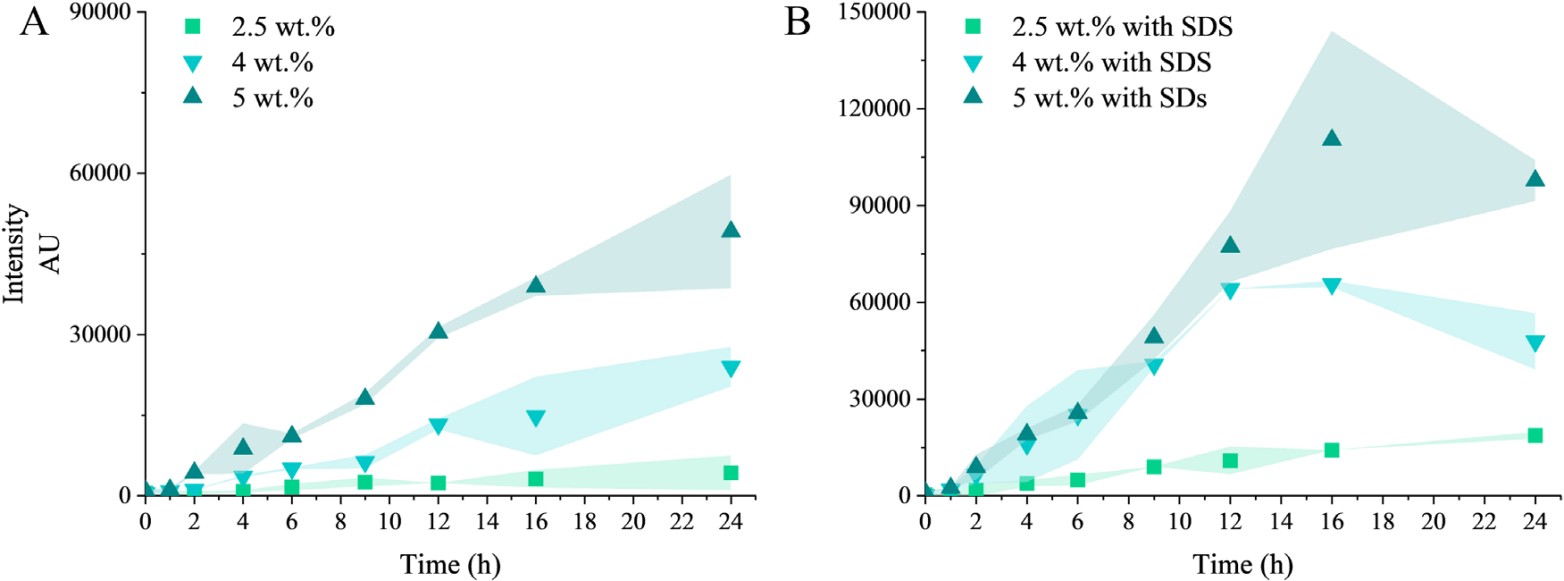
The fibrillization kinetics of ZP24 monitored by ThT‐binding fluorescence assay. A) ZP24 alone at different concentrations; B) fibrillization of ZP24 with SDS as an additive. The fibrillization was carried out at pH 8, 60 °C. The shadow along the plots indicates the standard deviation of the data.

The formation of fibrillar aggregates during the heat treatment (60 °C) of zein hydrolysate ZP24 was monitored by an increase in ThT fluorescence signal. At a low weight percentage (2 wt.%), a gradual increase in ThT signal was observed over 72 h (**Figure** **3**A,B). Birefringence appeared in the fibrillization mixture during the early stages (4–6 h), but prolonged heating time led to a turbid solution with precipitation. To prevent the formation of large aggregates and to regulate the assembly process, surfactants were added. Among the investigated surfactants, the inclusion of sodium dodecyl sulfate (SDS) resulted in a sixfold increase in ThT signal, indicating its suitability for enhancing the efficiency of fibril assembly from zein peptides (Figure 3B). Besides birefringence under polarized light, this sample exhibits characteristic UV spectrum upon binding with Congo red corresponding to amyloidal aggregates (Figure S3, Supporting Information).^[^ ^34^ ^]^ Transmission electron microscopy (TEM) confirmed fibrillar aggregates (Figure 3C–F). ZP24 alone formed straight filaments with negligible twisting (Figure 3C), while the addition of surfactants yielded distinct morphologies. Twisted fibrils were observed with SDS (Figure 3D); whereas thick, tube‐like fibrils and short, bundled needle‐like fibrils formed for systems with Span 80 (Figure 3E) and Tween 20 (Figure 3F), respectively. Previous investigations have shown that anionic surfactants modulate β‐sheet structure formation and affect the morphology of protein fibrillate aggregate below their critical micelle concentration.^[^ ^35^, ^36^ ^]^ Anionic concentration plays a crucial role in protein fibrillization by interplaying with the electrostatic and hydrophobic forces between the monomers.^[^ ^37^, ^38^ ^]^ Fibrillization kinetics were also influenced by the concentration of ZP24. Increasing ZP24 concentration shortened the lag time from 4 h at 2.5 wt.% to 1 h at 5 wt.%, with fibril formation intensifying accordingly (**Figure** **4**A). Additionally, 2.5 mm (0.072 wt.%) SDS accelerated fibrillization across all the ZP24 concentrations investigated (Figure 4B). At concentrations of 4 and 5 wt.%, fibril formation plateaued after 16 h, followed by a decline.

**Figure 5:**
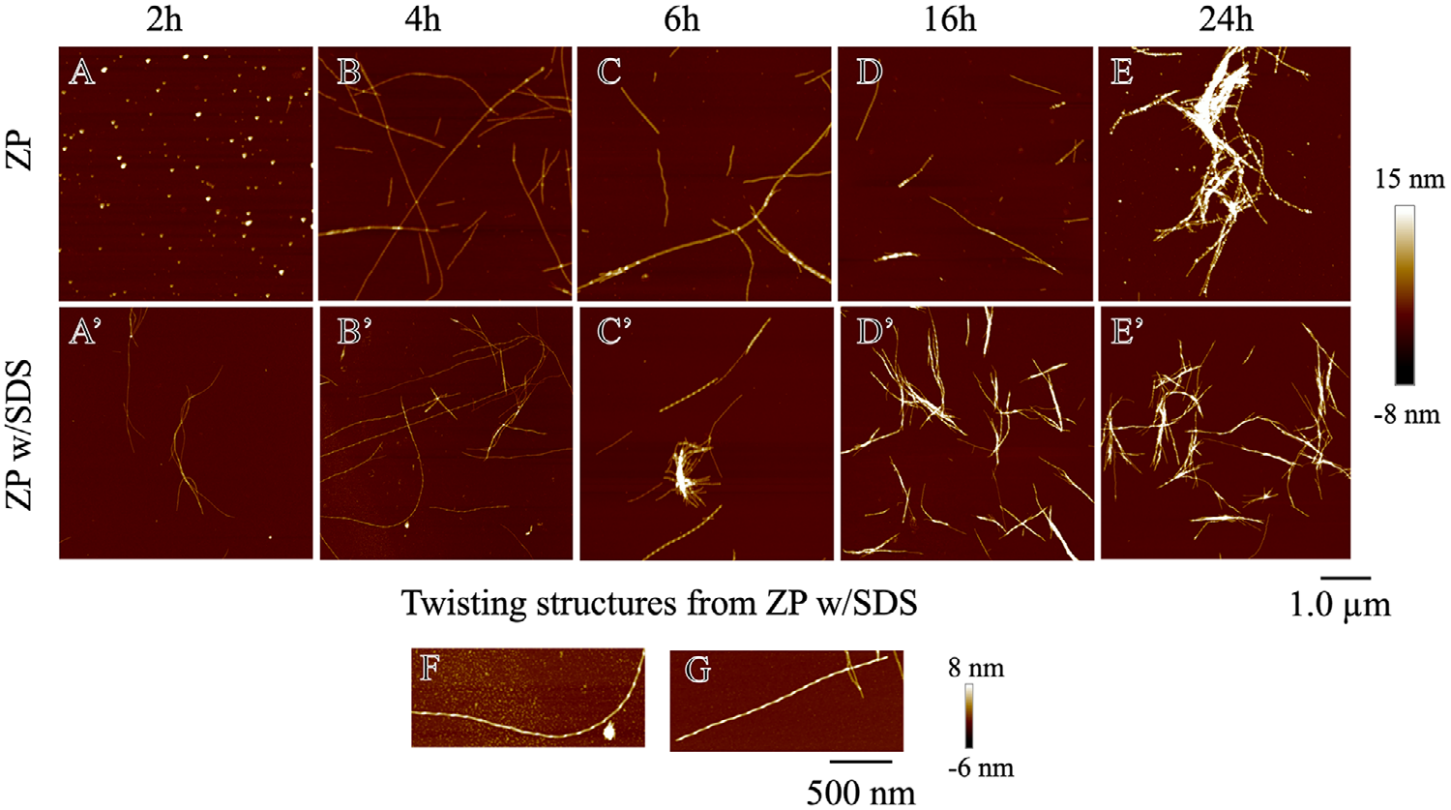
Morphological analysis by AFM of zein nanofibrils over time. A–E) nanofibrils from the system with ZP24 alone; A’–E’ nanofibrils from the system of ZP24‐SDS; F,G) shows the details of the twisting structure from ZP w/SDS at 4 and 6 h of incubation, respectively.

**Figure 6:**
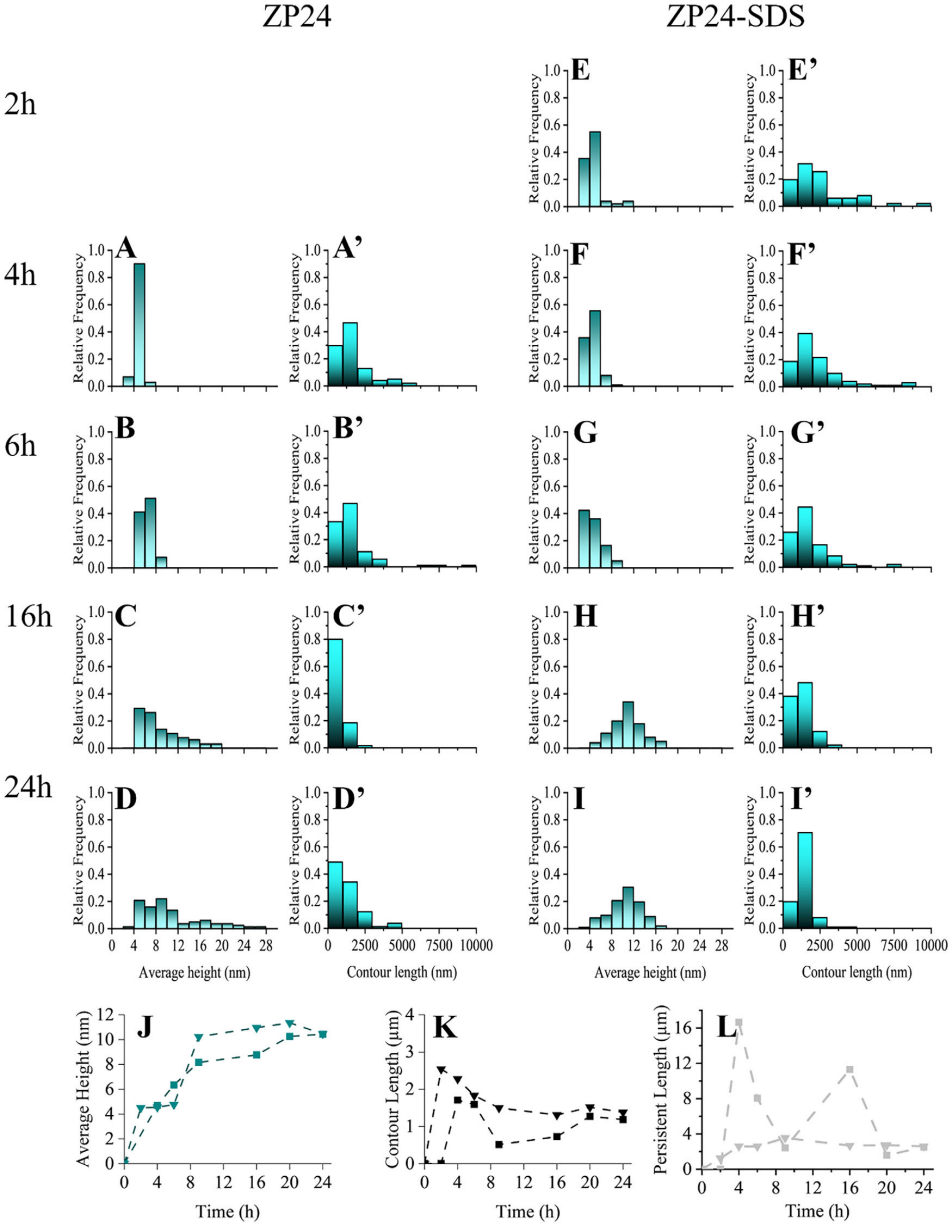
Zein nanofibril morphological analysis by FiberApp. A–I) change in average height over time A’–I’) change in contour length over time. The distribution graphs were processed based on data from 50–100 fibril counts. J–L) Change in average height, contour length and persistence length over time; triangular and square symbols represent pure ZP and ZP with SDS as additive, respectively.

Atomic force microscopy (AFM) analysis revealed time‐dependent changes in zein nanofibril morphology (**Figures** **5** and 6). Initially, globular aggregates formed at 2 h (Figure 5A), with single stranded fibrils appearing at 4 h (Figure 5B). Between early stages of 4–6 h, long (≈70% > 2 µm) and rigid fibrils (persistent length> 8 µm) dominated (Figures 5B,C and **6**A,B,A’,B’,L). Over time, the fibrils shortened due to continuous shearing and thickened by attaching to each other, and formed short fibril bundles, leading to a heterogeneous system, with most fibrils measuring < 1 µm by 24 h and varying in height from 4 to 28 nm (Figure 6D,D’). The presence of SDS induced curvature in thread‐like fibrils as early as 2 h (Figure 5A’), with twisted structures becoming more prominent at 4 and 6 h (Figure 5B’,C’). These nanofibrils were long, thin, and semi‐flexible, with periodic twisting and an average height between 2–6 nm (Figure 6F,G), which is similar to those semiflexible nanofibrils originating from oat globulin and β‐lactoglobulin.^[^ ^39^, ^40^ ^]^ At late‐stage incubation (>12 h), unlike the fibrils formed in the pure ZP24 system, those formed with SDS appeared as multi‐stranded fibrils, likely due to hierarchically intertwining between protofilaments.^[^ ^41^ ^]^ They were also shortened, possibly by shear forces, into needle‐like structures (Figures 5D’,E’, and 6K). After 16 h, fibrils stabilized at an average height of 10.47 ± 2.8 nm and a length of 1.38 ± 0.54 µm (Figure 6H,I,H’,I’), with rigidity remaining constant (≈2 µm) throughout the process (Figure 6L). Fibrillization processes showed inverse power‐law behavior in the fibril length decay overtime starting from 4 h (Figure 6K). The stability against fragmentation was predicted by the asymptotic line of a log–log plot of average length versus time.^[^ ^42^ ^]^ Result indicated that both types of zein fibrils have similar dynamic stability under long‐time shearing, with pure ZP24 fibrils slightly more stable than those with SDS (Figure S4, Supporting Information). As SDS contributed to better fibrillization efficiency and fibril morphology homogeneity within 24 h of incubation, nanofibrils from the ZP24‐SDS system at 6 h (F6H) and 24 h (F24 h) were selected for further structural and functional characterization.

### 2.4 Structural Characterization

**Figure 7:**
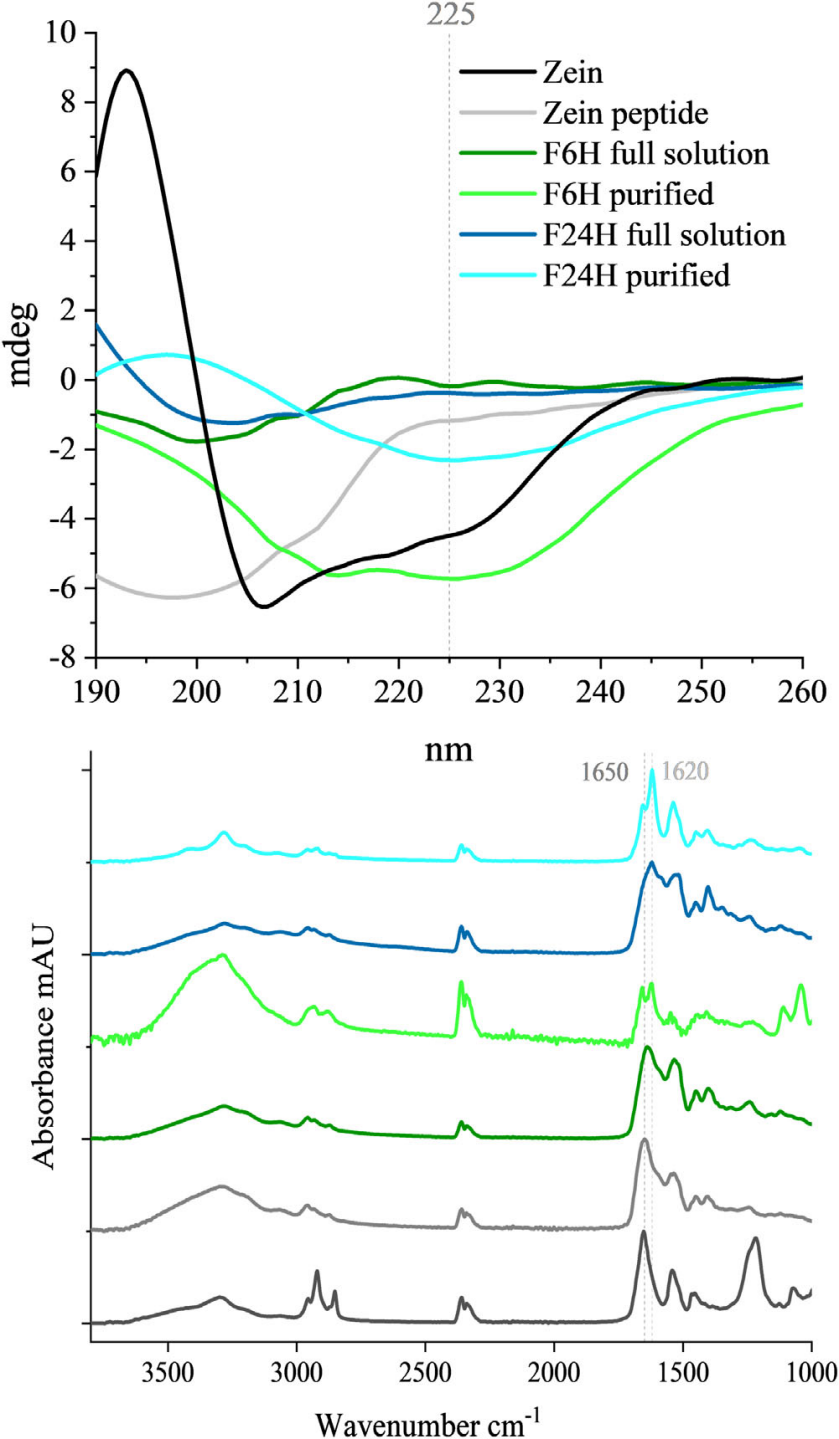
Secondary structure of zein nanofibrils. Zein fibrils formed at early stage (F6H) and mature fibrils (F24H) were analyzed by circular dichroism (A) and Fourier transform infrared spectroscopy (B). Crude fibril samples in full solution and purified zein fibrils from retentate upon ultrafiltration were compared with native zein and zein peptide.

Secondary structural changes accompanying fibril formation were investigated using CD and FTIR. Native zein exhibited a CD spectrum with a negative minimum at ≈207 nm and a positive peak between 190 to 200 nm simultaneously, along with strong absorption at 1650 cm^−1^ in the FTIR spectrum (**Figure** **7**), which is indicative of its predominantly α‐helical structures.^[^ ^43^ ^]^ In contrast, peptides from pepsin hydrolysis of zein showed a CD spectrum with a negative peak ≈200 nm, which is characteristic of random coil structures. The CD spectrum of purified mature fibrils (F24H) showed a broad negative peak at ≈225 nm, signifying rich β‐sheet structures, similar to nanofibrils derived from lysozyme hexapeptides.^[^ ^44^ ^]^ These authors attributed such CD spectral feature to needle‐like β‐sheet assemblies from short peptides containing isoleucine and glutamine. The early‐stage fibrils (F6H) displayed an incomplete structural transformation, as indicated by a broad negative peak between 210 and 225 nm (Figure 7A). In the FTIR spectra, the amide I region (1600–1700 cm^−1^), sensitive to hydrogen bonding, revealed a shift from 1650 to 1620 cm^−1^ during fibrillization, indicating the formation of an intermolecular β‐sheet structure (Figure 7B). Similar shifting of a major FTIR peak from ≈1650 to ≈1630 cm^−1^ was observed in zein nanofibrils formed in an ethanol‐aqueous (70/30 v/v) environment.^[^ ^18^ ^]^ Our zein nanofibrils, synthesized in purely aqueous conditions, may involve stronger hydrogen bonding interactions, reflected by a lower wavenumber for the β‐sheet structure.^[^ ^45^ ^]^ According to these authors, the phenomenon of forming lower‐wavenumber intermolecular β‐sheet at the expense of a random coil/α‐helical structure in a variety of systems was attributed to amyloid fibril formation. Other spectral changes including the disappearance of absorption signals of N─H stretching at ≈3200 cm^−1^ and ‐CH_2_ stretching at ≈2900 cm^−1^,^[^ ^46^ ^]^ is possibly indicative of intensified hydrophobic interaction and H‐bonding caused by fibrillization.^[^ ^47^ ^]^ Our results suggest that the synthesized zein nanofibrils exhibit amyloidal β‐sheet structures.

**Figure 8:**
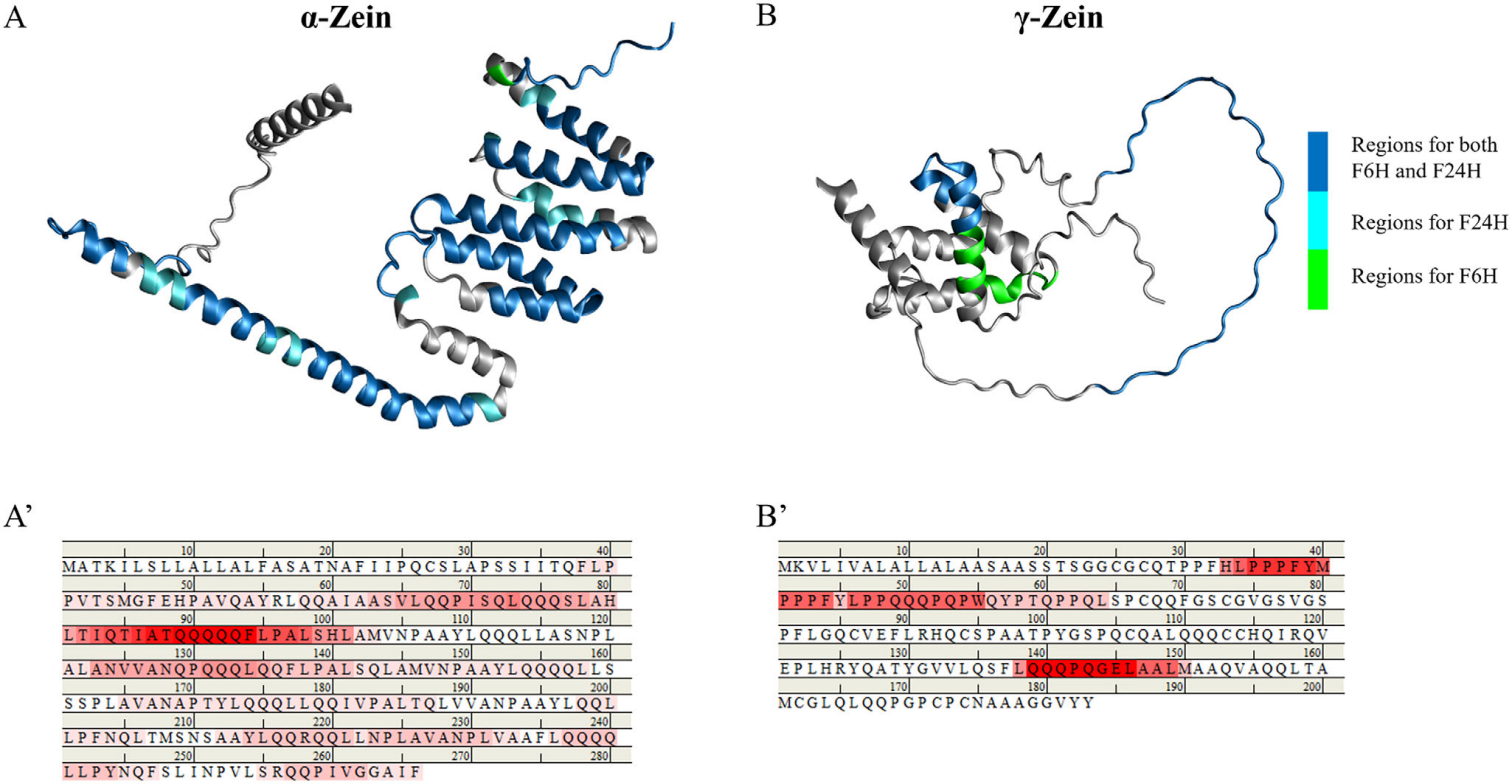
Amyloidogenic fragments on α‐zein and γ‐zein. Amyloidogenic peptides identified by LC‐MS/MS were highlighted on the molecules (A and B) or sequences (A’ and B’). The different shades of red along zein sequences represent normalized frequency based on mass spectra counts. The 3D structure of α‐zein (UniProt accession number: O48966) and γ‐zein (UniProt accession number: P08031) were generated by Alphafold.

To identify the fibrillization‐prone peptide segments of zein, peptide mass fingerprinting was performed on purified F6H and F24H nanofibrils upon chymotryptic digestion. The result showed that these nanofibrils were predominantly composed of peptide fragments from α‐zein, with a minor portion from γ‐zein, comprising 89 and 15 peptide fragments, respectively (Tables S1 and S2, Supporting Information). These peptide fragments covered more than 50% of the α‐zein sequence, with slightly more peptide fragments detected in the 24‐h sample than in the 6‐h sample. The location of these peptides on the two zein molecules is depicted in **Figure** **8**A,B. In addition, all detected peptide sequences are considered as amyloidogenic segments of zein, which are highlighted in different shades of red along the sequence α‐zein (Figure 8A’) and γ‐zein (Figure 8B’). The α‐zein region from the location from residue 79 to 101 AHLTIQTIATQQQQQFLPALSHL and the γ‐zein region from residue 33 to 64 HLPPPFYMPPPFYLPPQQQPQPWQYPTQPPQL are considered hotspots for fibrillization due to their high amyloidogenic propensity. Two major characteristics were observed among the amyloidogenic peptides of zeins: 1) a high proportion of multiple glutamine (QQQQ_n_) structure; and 2) the frequent presence of phenolic hydrophobic side chain from phenylalanine (F), tyrosine (Y), and tryptophan (W). Hydrophobic side chains provide a thermodynamic advantage in self‐assembly of the peptides, whereas, amide sidechains from glutamine ensure tight β‐sheet packing through hydrogen bonding.^[^ ^37^ ^]^ The amino acid sequence of zein, particularly α‐zein, makes it an ideal starting material for fibrillization. We observed that once abundant fibrillization‐prone segments, as described above, were released into an aqueous environment, they actively participated in nanofibril formation. It was also shown that some peptide segments were found uniquely in 6 and 24 h fibrillization samples, respectively (Tables S1 and S2, Supporting Information). In F6H, these included QIRQVEPL and MAAQVAQQL, both from γ‐zein; whereas, in F24H, these included QQAIAAS, QQSLA, and QQLLPFNQL from α‐zein. These differences may account for the morphological variations observed between the F6H and F24H fibrils, as discussed in section 2.3 (Figure 5C’ vs Figure 5E’).

### 2.5 Functionality Assessment

**Figure 9:**
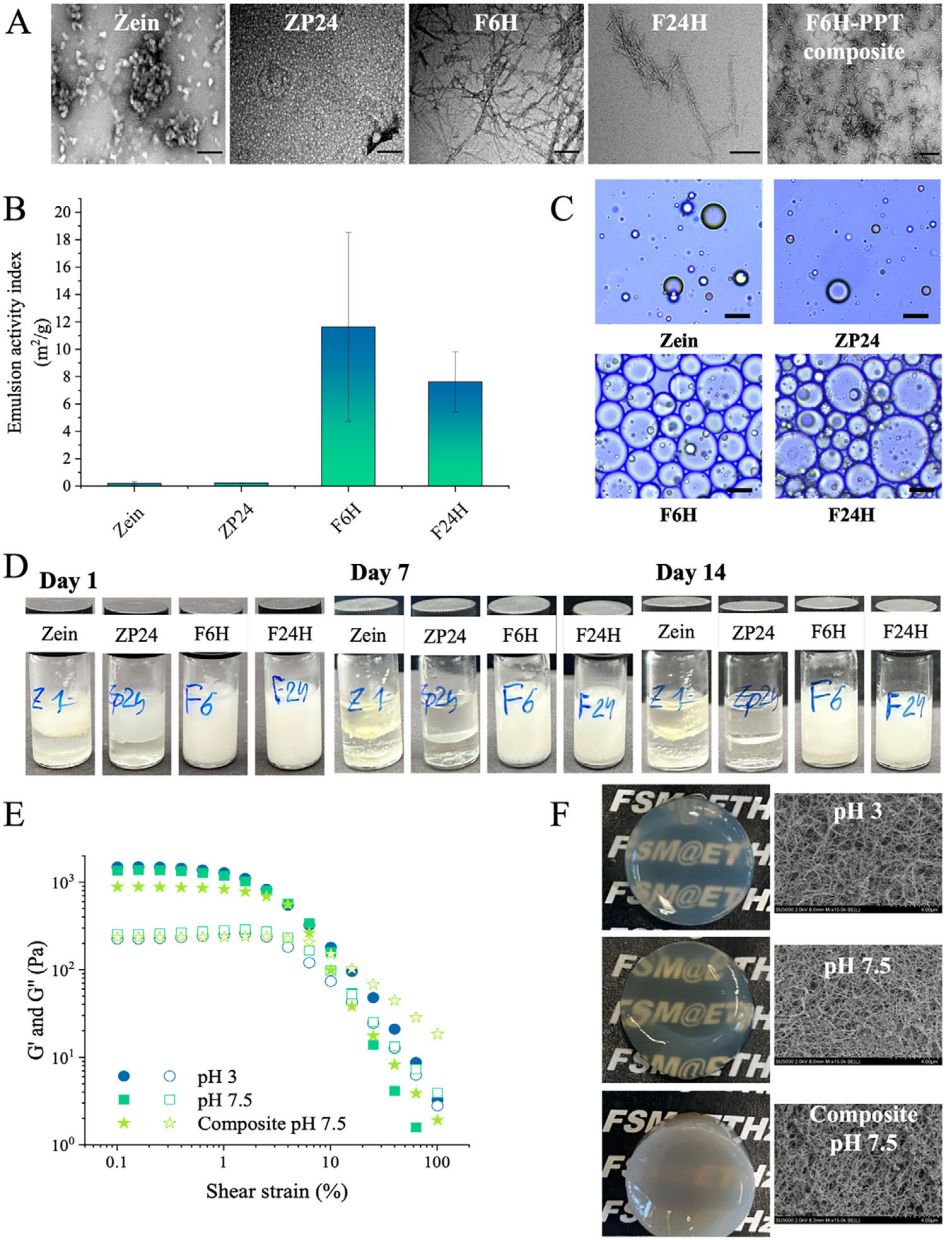
Functionality assessment of different zein materials. A) TEM images of zein materials for functional analysis, the scales represent 200 nm; B–D) emulsifying properties: native zein, zein peptide ZP24, and zein nanofibrils F6H and F24H were evaluated for their emulsifying activity index (B). C,D) Micro‐images of emulsion droplets, the scale bars represent 50 µm (C) and visual inspection of the emulsion over 2 weeks (D); E,F) gelling properties: amplitude sweep (E) and macroscopic and microscopic morphology (F) of the hydrogel of zein and zein‐potato hybrid nanofibrils. PPT refers to potato protein.

Recent findings have demonstrated that amyloid fibrils from food origin are safe nutrition ingredients.^[^ ^48^ ^]^ To reveal the potential of current fibrillization process in deriving functional ingredients from zein, we studied two functionalities, interfacial stabilization and gelation behaviors, that are important for proteinaceous ingredients in food, pharmaceutical or cosmetic formulations. The functionality of protein colloids is generally dictated by their microstructures. The microstructures of related zein materials were captured by TEM, as shown in **Figure** **9**A.

Protein materials are often used as natural emulsifiers due to their surface activity and amphiphilicity. A high‐internal‐phase emulsion with a 75% volume fraction of oil as the dispersed phase was produced using different zein materials. Neither zein nor ZP24 peptide mixture was able to create a stable emulsion system, resulting in immediate phase separation upon the application of shear. In contrast, nanofibrils exhibited superior emulsifying ability to native zein or zein peptides. The zein nanofibrils showed an emulsifying activity index (EAI) ranging from 8 to 12 m^2^ g^−1^, with a higher EAI observed for early‐stage fibrils (F6H) than for mature fibrils (F24H) (Figure 9B). The emulsion prepared using nanofibrils remained stable over 2 weeks, with minimal phase separation (Figure 9D). As shown in the micro‐images, the nanofibrils formed a thick interface surrounding the oil droplets, which allowed tight packing between the oil droplets and ensured emulsion stability (Figure 8C). The superior emulsifying ability of the nanofibrils in stabilizing high‐internal‐phase emulsions is ascribed to their molecular entanglement and the formation of a cohesive viscoelastic interface.^[^ ^49^ ^]^ In contrast, emulsion droplets were hardly visible when native zein or zein peptides were used as emulsifiers. Native zein is insoluble in both water and oil phase, while ZP24 peptides are too small in molecular size to form stable interfaces, which explains their poor emulsifying properties. Previous research has reported that structural modification is essential for native zein to create a stable emulsion system. Classical antisolvent precipitation has been used to form nanoscale zein colloidal particles to support the fabrication of Pickering‐type emulsions.^[^ ^50^, ^51^ ^]^ Moreover, gel‐like emulsion can be achieved using protein composites containing zein prepared via enzymatic cross‐linking.^[^ ^52^ ^]^ To the best of the authors’ knowledge, this study is the first to report the emulsifying properties of zein nanofibrils.

Due to their high aspect ratio, rod‐like structure, unique molecular surface, and high structural stability, protein nanofibrils are promising building blocks for functional hydrogels and aerogels, as well as gelling and thickening reagents.^[^ ^2^, ^32^ ^]^ We investigated the gelation behavior of zein nanofibrils. Gelation was induced by 100 mm CaCl_2_ at both pH 3 and 7 for F6H and F24H nanofibrils. It was found that F6H formed a relatively soft and fragile paste (G’ from ≈360 to ≈460 Pa), lacking a 3D structure, likely due to the lower concentration of fibril at the early stage of fibrillization (Figure S5, Supporting Information). Previous investigations have demonstrated a positive correlation between fibril concentration and gel strength (G’).^[^ ^53^ ^]^ In contrast, the mature F24H nanofibrils formed a transparent self‐supporting structure at both pH 7.5 and 3 (Figure 9F). Amplitude sweeps indicated a viscoelastic solid‐like consistency for these hydrogels, with similar linear viscoelastic regions and a G’ of over 10^3^ Pa (Figure 9E). SEM micro‐images revealed fibrous structures for both hydrogels, with no significant differences observed between the two gelling conditions (Figure 9F).

Amyloid fibrils are known for their seeding ability, which refers to the acceleration of the fibrillization process by adding a pre‐formed protein nanofibrils to a protein solution.^[^ ^3^ ^]^ F6H nanofibrils were successfully used to seed potato proteins (PPT) at a weight ratio of 1:1, producing shorter, wormed‐like composite fibrils within 1 h of treatment at pH 8 and 60 °C (Figure 9A). Compared to the 6 h required for potato protein fibrillization reported in a previous study,^[^ ^22^ ^]^ the appearance of fibrils via seeding occurred very rapidly. The composite also formed hydrogel at pH 7.5 with 100 mm CaCl_2_, which appears as opaque and in a beige color. Compared to F24H hydrogel, the hybrid potato‐zein fibrils exhibited a relatively lower G’, which indicates a decrease in gel strength. The SEM image showed that the hybrid hydrogel was constructed by a slightly thicker fibrous network that resembled a porous network. As potato protein and zein complement each other in amino acid profile, this composite nanofibril may offer additional advantages in nutritional quality if used as a functional ingredient. We also observed that all three types of hydrogels were stable upon solvent exchange from an aqueous environment to ethanol. Indeed, An et al. (2016) reported similar hydrogel stability for α‐zein nanofibrils prepared in an ethanol‐water binary system, and attributed this to the excellent mechanical property of β‐sheet structure‐based nanofibrils.^[^ ^18^ ^]^

The present protease‐assisted process made possible to functionalize zein into emulsifiers or building blocks for fibrous networks, which may find many applications in food, materials and biomedicine fields. The feasibility of fibrillization in a purely aqueous environment for other food proteins has been already demonstrated at an industrial scale, as shown by successful application in converting soy whey proteins into biodegradable and bio‐based packaging materials.^[^ ^54^ ^]^ Thus, the protease‐assisted synthesis of zein nanofibrils, which eliminates the need for organic solvents and enables targeted hydrolysis, may offer significant advantages for large‐scale production of this class of amyloid fibrils and enable the valorization of proteinaceous side streams from corn processing.

## 3 Conclusion

In the present study, a pepsin‐mediated bioprocess to synthesize zein nanofibrils has been successfully established, significantly reducing reliance on organic solvents which are typically essential in conventional methods of zein fibrillization. This novel approach overcomes the solubility and structural limitations of zein by transforming it into nanofibrils that are dispersible in a pure aqueous environment. These nanofibrils exhibit exceptional interfacial stabilization in high‐internal‐phase emulsions and the ability to form fibrous hydrogels. Assembled primarily from peptide segments from α‐zein (comprising more than 50% of the protein sequence), the nanofibrils are semiflexible with a protofilament intertwining morphology like functional amyloid fibrils derived from other food proteins, thus confirming their potential as valuable functional materials. Given the abundance of α‐zein in corn biowaste from industrial side streams, this study presented a promising approach for valorization of corn biowaste into functional building blocks. Future studies should focus on the optimization of the enzymatic reaction to avoid lyophilization‐concentration step, to improve enzyme reusability and to ensure the industrial scalability of the current bioprocess for zein nanofibrils production. The additional functionalities of these zein nanofibrils will also be explored to develop applications across various fields.

## Conflict of Interest

The authors declare no conflict of interest.

## Supporting information



Supporting Information

## Data Availability

The data that support the findings of this study are available from the corresponding author upon reasonable request.
