# Supplementary material for: Protease‐Mediated Synthesis of Zein Nanofibrils: From Structural Elucidation to Functional Application
Source: Adv Sci (Weinh). 2025 Mar 31;12(21):2414606. doi: 10.1002/advs.202414606 (PMC12140339; doi:10.1002/advs.202414606)
Supplement: Supplementary file 1 — Supporting Information [file ADVS-12-2414606-s001.docx]

**Supporting information**

**Protease-mediated Synthesis of Zein Nanofibrils: from Structural Elucidation to Functional Application**

Mingqin Li^1*^, Tonghui Jin^1^, Simone Wüthrich^2^, Qiyao Sun^1^, Ting Li^3^, Jiangtao Zhou^1^, Zhou Dong^1^, Eva Maria Zunzuneigui Bru^1^, and Raffaele Mezzenga^1,4*^

1. Department of Health Sciences and Technology, ETH Zurich, Schmelzbergstrasse 9, 8092 Zurich, Switzerland
2. Functional Genomics Center Zurich UZH/ETH Zurich, Winterthurestrasse 190, 8057 Zurich, Switzerland
3. School of Food Science and Technology, Jiangnan University, Lihu Road 1800, Wuxi, 214122, China
4. Department of Materials, ETH Zurich, Wolfgang-Pauli-Strasse 10, 8093 Zurich, Switzerland

* Correspondence:

Mingqin Li: mingqin.mingqinli@hest.ethz.ch

Raffaele Mezzenga: raffaele.mezzenga@hest.ethz.ch


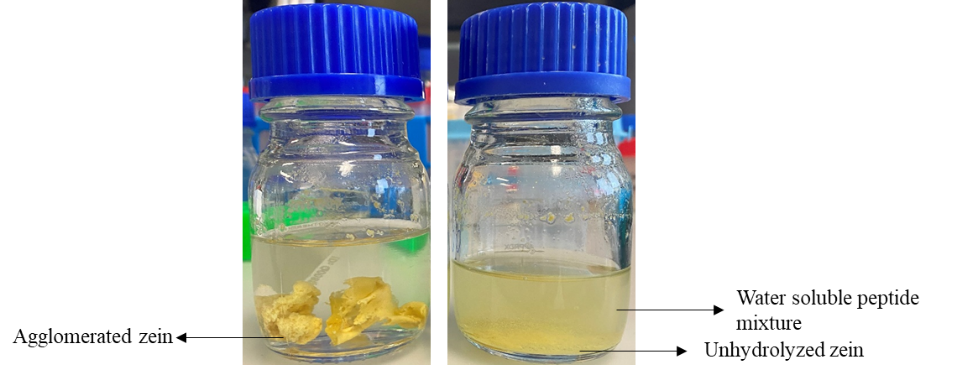


**Figure S1.** Heat treatments of zein at 60°C alone (left) and with enzyme (right).


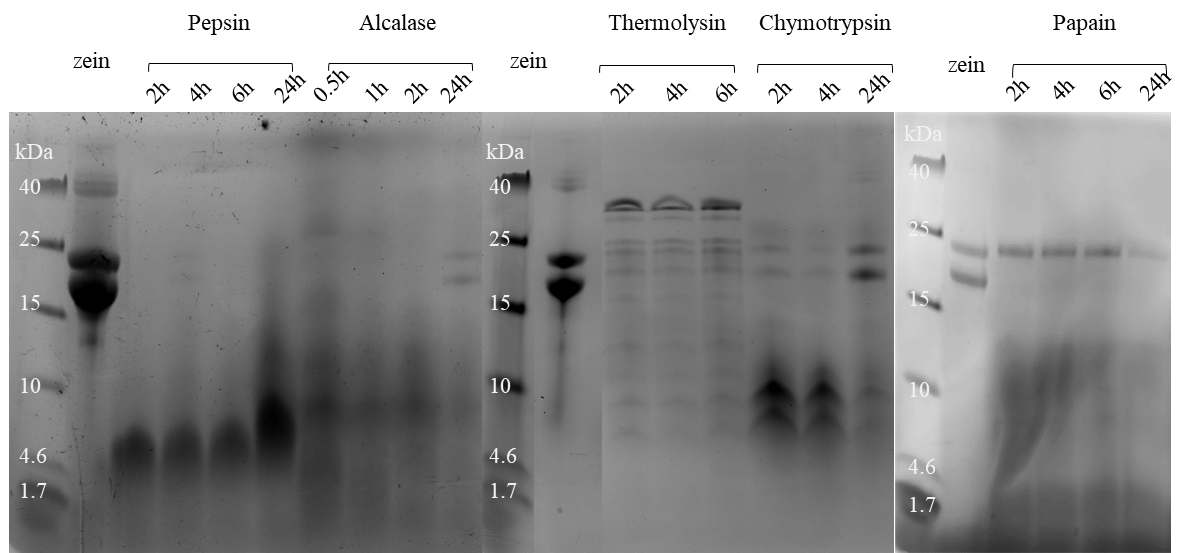


**Figure S2.** Tricine-SDS-PAGE analysis of zein and zein hydrolysates obtained from enzymatic hydrolysis over time.


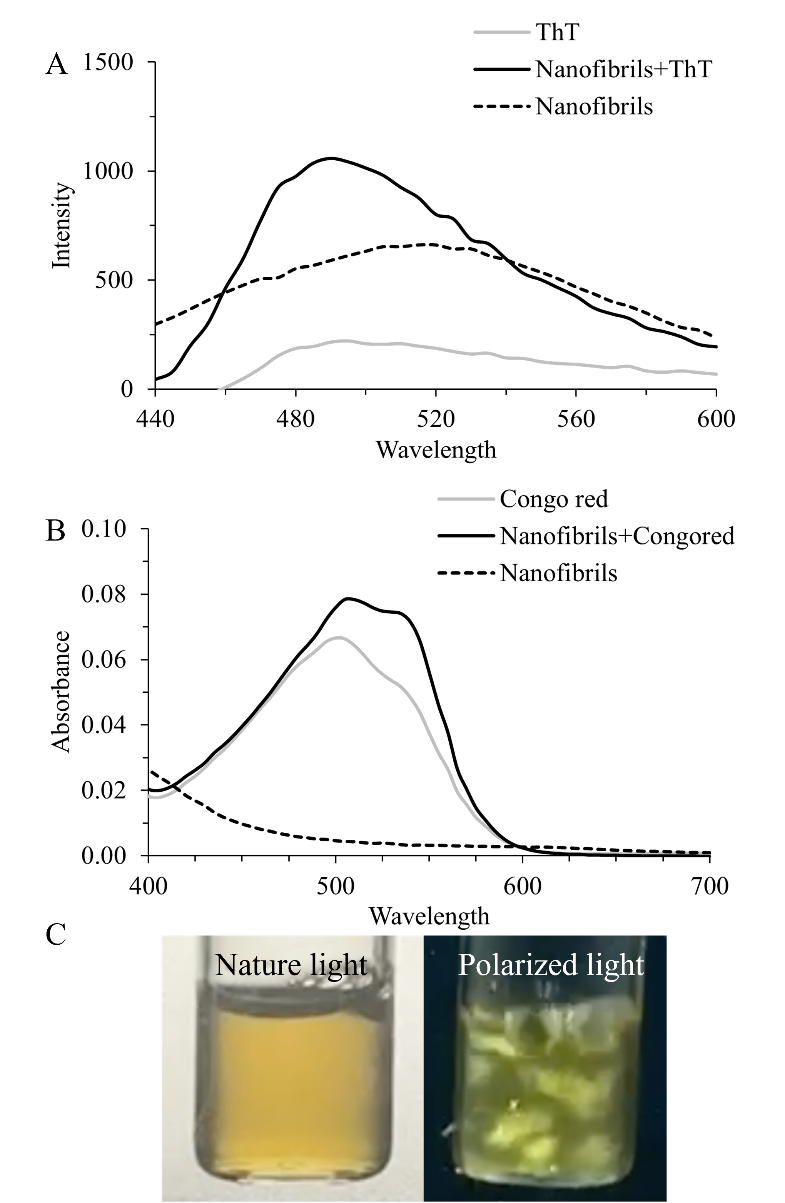


**Figure S3.** Spectral characteristics and birefringence of ZP24 upon fibrillization. A. Emission spectrum of ThT upon binding with fibrillized ZP24 at excitation wavelength of 420 nm; B. Absorbance spectrum of Congo red upon binding with fibrillized ZP24; C. Appearance of fibrillized ZP24 solution under nature light or polarized light.


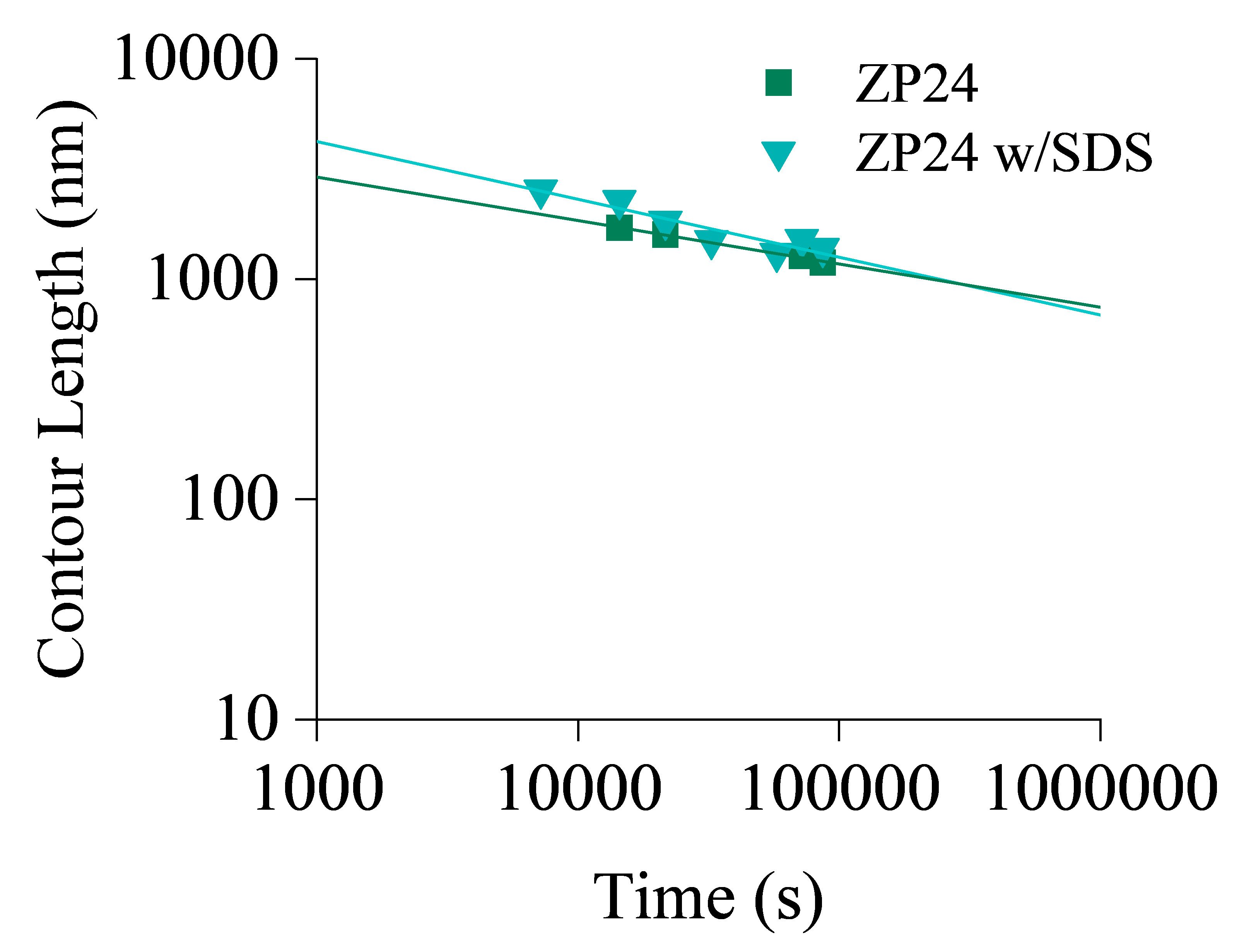


Figure S4. Linear fitting in log-log plot of average contour length of zein nanofibril versus time.

**Figure S5.** Oscillatory amplitude sweeps of hydrogel from F6H samples.

**Table S1.** Fibril-forming peptides identified via proteomic analysis matched with α-zein (Accession No. O48966).

| *Fibril genic peptides* | | | | |
| --- | --- | --- | --- | --- |
| **Sequence** | **Modification** | **Predicted mass** | **ppm** | **Location** |
| FLPPVTSMGF | Met oxidation peptide | 1110.54 | * | 38-47 |
| LPPVTSMG | Met oxidation peptide | 800.41 | * | 39-46 |
| EHPAVQAY |  | 913.43 | * | 48-55 |
| SVLQQPISQL |  | 1111.62 | ** | 64-73 |
| AHLTIQTIATQQQQQF | Deamidation fragment detected at 24 h sample | 1854.96 | *** | 79-94 |
| AHLTIQTIATQ |  | 1195.66 | * | 79-89 |
| LTIQTIATQQQQQF |  | 1646.86 | * | 81-94 |
| TIQTIATQQQQQFLPAL |  | 1928.04 | * | 82-98 |
| TIQTIATQQQQQFLPALSHL | Deamidation fragment detected at 24 h sample | 2265.21 | * | 82-101 |
| TIQTIATQQQQQFLPALSH |  | 2152.13 | * | 82-100 |
| TIQTIATQQQQQFL |  | 1646.86 | * | 82-95 |
| TIQTIATQQQQQF | Deamidation fragment detected | 1533.78 | * | 82-94 |
| TIQTIATQQQ |  | 1130.59 | * | 82-91 |
| TIQTIATQQQQ |  | 1258.65 | * | 82-92 |
| TIQTIATQQQQQ |  | 1386.71 | * | 82-93 |
| IQTIATQQQQQF |  | 1432.73 | * | 83-94 |
| QTIATQQQQQF |  | 1319.65 | * | 84-94 |
| TIATQQQQQF |  | 1191.59 | * | 85-94 |
| IATQQQQQFLPAL |  | 1484.80 | * | 86-98 |
| IATQQQQQFLPALSHL | Deamidation fragment detected at 24 h sample | 1821.97 | * | 86-101 |
| IATQQQQQF |  | 1090.54 | * | 86-94 |
| TQQQQQFLPALSHL |  | 1637.85 | * | 88-101 |
| QQQQQFLPAL |  | 1199.63 | * | 89-98 |
| QQQQQFLPALSHL |  | 1536.80 | * | 89-101 |
| QQFLPALSHL |  | 1152.63 | ** | 92-101 |
| QFLPALSHL |  | 1024.57 | * | 93-101 |
| ANVVANQPQQQLQQFLPAL |  | 2106.12 | * | 123-141 |
| ANVVANQPQQQLQQFL |  | 1824.95 | * | 123-138 |
| ANVVANQPQQQL |  | 1308.68 | ** | 123-134 |
| ANVVANQPQQQLQ |  | 1436.74 | * | 123-135 |
| QLQQFLPAL |  | 1056.60 | ** | 133-141 |
| QQFLPALSQL |  | 1143.63 | * | 135-144 |
| AMVNPAAYLQQQQL | Met oxidation peptide | 1573.79 | * | 145-158 |
| AVANAPTYLQQQL |  | 1415.74 | * | 165-177 |
| AVANAPTYL |  | 918.48 | * | 165-173 |
| QQQLLQQIVPAL |  | 1377.80 | * | 174-185 |
| LQQIVPALTQ |  | 1109.64 | * | 178-187 |
| AYLQQRQQL |  | 1146.61 | * | 213-221 |
| YLQQRQQL |  | 1075.58 | * | 214-221 |
| LNPLAVANPL |  | 1020.60 | * | 222-231 |
| NPLAVANPLVA |  | 1077.62 | * | 223-233 |
| NPLAVANPL |  | 907.51 | * | 223-231 |
| LQQQQLLPY |  | 1129.61 | * | 236-244 |
| QQQQLLPY |  | 1016.53 | * | 237-244 |
| SRQQPIVGGAIF |  | 1271.70 | * | 255-266 |
| RQQPIVGGAIF |  | 1184.67 | * | 256-266 |
| QQPIVGGAIF |  | 1028.57 | * | 257-266 |

| *Unique fibril genic peptides found in 6 h samples* | | | | |
| --- | --- | --- | --- | --- |
| **Sequence** | **Modification** | **Predicted mass** | **ppm** | **Location** |
| TIQTIATQQQQQFLPALS |  | 2015.07 | * | 82-99 |
| TIQTIATQQ |  | 1002.53 | * | 82-90 |
| IQTIATQQQQQF |  | 1432.73 | * | 83-94 |
| QQQQFLPALSHL |  | 1408.75 | * | 90-101 |
| QQFLPALSQ |  | 1030.54 | ** | 135-143 |
| VNPAAYLQQQQL |  | 1371.71 | * | 147-158 |
| QQIVPALTQ |  | 996.56 | * | 179-187 |
| LSRQQPIVGGAIF |  | 1384.78 | * | 254-266 |
| SRQQPIVGGAI |  | 1124.63 | * | 255-265 |
| SRQQPIVGGA |  | 1011.55 | * | 255-264 |
| *Unique fibril genic peptides found in 24 h samples* | | | | |
| **Sequence** | **Modification** | **Predicted mass** | **ppm** | **Location** |
| QQAIAASVL |  | 899.51 | * | 58-66 |
| ASVLQQPISQLQQQSL |  | 1766.95 | * | 63-78 |
| ASVLQQPISQL |  | 1182.66 | * | 63-73 |
| SVLQQPISQLQQQSL |  | 1695.92 | * | 64-78 |
| VLQQPISQLQQQSL |  | 1608.88 | * | 65-78 |
| LQQPISQLQQQSL |  | 1509.82 | * | 66-78 |
| QQPISQLQQQSLAHL |  | 1717.91 | * | 67-81 |
| QQPISQLQQQSL | Deamidation fragment also detected | 1396.73 | ** | 67-78 |
| QQPISQLQQQ |  | 1196.62 | * | 67-76 |
| QPISQLQQQSL |  | 1268.67 | * | 68-78 |
| PISQLQQQSLAHL |  | 1461.79 | * | 69-81 |
| LQQQSLAHL |  | 1036.57 | * | 73-81 |
| TIQTIATQQQQQFLP |  | 1743.92 | * | 82-96 |
| TIQTIATQQQQQFLPA |  | 1814.95 | * | 82-97 |
| IQTIATQQQQQFLPAL |  | 1826.99 | ** | 83-98 |
| IQTIATQQQQQFLPALSHL |  | 2164.16 | * | 83-101 |
| ATQQQQQFLPAL |  | 1371.71 | * | 87-98 |
| ATQQQQQFLPALSHL |  | 1708.89 | * | 87-101 |
| QQQFLPALSHL |  | 1280.69 | * | 91-101 |
| LPALSHLAM | Met oxidation peptide | 967.52 | * | 95-103 |
| ALANVVANQPQQQL |  | 1492.80 | * | 121-134 |
| ALANVVANQPQQQLQ |  | 1620.86 | *** | 121-135 |
| ANVVANQPQQQ |  | 1195.59 | * | 123-133 |
| NVVANQPQQQLQQF |  | 1640.83 | * | 124-137 |
| NVVANQPQQQL |  | 1237.64 | * | 124-134 |
| QQQLQQFLPAL |  | 1312.71 | * | 131-141 |
| QQLLPFNQL |  | 1099.60 | * | 198-206 |
| AAYLQQRQQL |  | 1217.65 | * | 212-221 |
| QQRQQLLNPL | Deamidation fragment also detected | 1236.69 | ** | 216-225 |
| NPLAVANPLV |  | 1006.58 | * | 223-232 |
| QQQQLLPYNQF |  | 1405.70 | * | 237-247 |
| SRQQPIVGG |  | 940.51 | * | 255-263 |

-The highlighted segments in cyan represent the regions uniquely found in F24H

-*, **, and *** represent the absolute deviation value < 1 ppm, 2-3 ppm, and 3-4 ppm, respectively

**Table S2.** Fibril-forming peptides identified via proteomic analysis matched with γ-zein (Accession No. P08031)

| *Unique fibril genic peptides found in 6 h samples* | | | | |
| --- | --- | --- | --- | --- |
| **Sequence** | **Modification** | **Predicted mass** | **ppm** | **Location** |
| YMPPPFY | Met oxidation peptide | 929.3993 | * | 39-45 |
| QIRQVEPL |  | 981.5607 | ** | 116-123 |
| MAAQVAQQL | Met oxidation peptide | 974.4855 | * | 150-158 |
| *Unique fibril genic peptides found in 24 h samples* | | |  |  |
| **Sequence** | **Modification** | **Predicted mass** | **ppm** | **Location** |
| LPPPFYMPPPF |  | 1301.6519 | * | 34-44 |
| PPPFYMPPPF | Met oxidation peptide | 1188.5677 | * | 35-44 |
| LPPQQQPQPWQ |  | 1345.6779 | * | 46-56 |
| LQQQPQGELAAL |  | 1294.6881 | * | 138-149 |
| *Fibril genic peptides* | |  |  |  |
| **Sequence** | **Modification** | **Predicted mass** | **ppm** | **Location** |
| HLPPPFYMPPPF | Met oxidation peptide | 1438.7107 | * | 33-44 |
| HLPPPFY |  | 869.4435 | * | 33-39 |
| YLPPQQQPQPW |  | 1380.6826 | * | 45-55 |
| LPPQQQPQPW | Deamidation fragment detected at 24 h sample | 1217.6193 | * | 46-55 |
| QYPTQPPQL | Deamidation fragment detected at 24 h sample | 1070.5397 | * | 56-64 |
| LQQQPQGEL |  | 1039.5298 | * | 138-146 |
| QQQPQGELAALM | Met oxidation peptide | 1312.6445 | * | 139-150 |
| QQQPQGELAAL |  | 1181.604 | * | 139-149 |

-The highlighted segments in green represent the regions uniquely found in F6H

--*, **, and *** represent the absolute deviation value < 1 ppm, 2-3 ppm, and 3-4 ppm, respectively

**Experimental**

**1. Materials**

Zein, proteases (including pepsin from porcine liver, chymotrypsin, thermolysin, and papain), surfactants (including sodium dodecyl sulfate (SDS), Tween 20, and Span 80) and fluorescent probe Thioflavin T for fibrillization kinetics, Congo red, (3-Aminopropyl)triethoxysilane (APTS) for the surface modification of mica, and chemicals for SDS-PAGE (including tricine, β-mecaptoethanol, glycerols, and Tris-HCl), were purchased from Sigma Aldrich (Merck & Cie, Buchs, Switzerland). Protease alcalase 2.4 L was a gift from Novozyme (Novozyme Switzerland AG, Athens, Greece). Fluorescent probe ANS for surface hydrophobicity and Coomassie Brilliant Blue G250 (CBB G250) dye were purchased from Fisher Scientific (Fisher Scientific AG, Reinach, Switzerland). Canola oil was acquired from a local supermarket COOP.

**2. Zein hydrolysis by proteases**

The suspension of zein at a concentration of 10 mg/mL was treated with selected proteases at a concentration of 0.003-0.08:1 w/w (enzyme-to-zein ratio), using the optimal reaction pH and temperatures for the enzyme (as shown in Table S3). The hydrolysis reactions were performed at these conditions for 0.5 to 24 h. Sample aliquots were taken to measure the bioconversion and the peptide product profile over the course of reaction time. The proteases were inactivated by incubating the reaction mixture at 85 °C for 15 min. The hydrolysate, containing a mixture of peptide, was separated from the unhydrolyzed and insoluble residues by centrifugation at 14000 $\times$g for 20 min. The recovered supernatant was lyophilized at -50 °C, 0.5 mBar using Labconco bench-top lyophilizer (4.5 L).

**Table S3.** Enzyme concentration, pH, and temperature used in each protease-catalyzed hydrolysis reaction.

| Enzymatic reaction | pH | Temperature (°C) | Enzyme concentration (Enzyme-to-zein w/w) |
| --- | --- | --- | --- |
| Pepsin | 3 | 40 | 0.003:1 |
| Alcalase | 8 | 50 | 0.08:1 |
| Thermolysin | 8 | 60 | 0.017:1 |
| Chymotrypsin | 8 | 40 | 0.02:1 |
| Papain | 7.5 | 40 | 0.01:1 |

**3. Bioconversion yield and protein content**

The weight percentage of the lyophilized peptide product over the initial dry weight of zein in the reaction mixture was calculated as the bioconversion yield. The protein content of the peptide product was measured by Hatree-Lowry assay and using a total organic carbon/nitrogen analyzer (SHIMADZU Corporation, Kyoto, Kyoto, Japan).

**4. Product profiles**

The molecular weight profile of the recovered hydrolysate peptide mixtures over time was evaluated by Tricine-SDS-PAGE. Peptide samples (10-50 mg/mL) were mixed with sample buffer (200 mM Tris-HCl pH 6.8, 2% SDS, 40% glycerol, 0.04% of CBB G250, containing 2% of β-mercaptoethanol) at a volume ratio of 1:1, and incubated at 95°C for 10 min for denaturation. 20 μL of this mixture was loaded to 16.5% Criterion^TM^ Tris-Tricine precast gel with a dimension of 13.3 × 8.7 cm (width × length) (Bio-Rad, Hercules, CA, U.S.A.). 10 μL of Spectra™ Multicolor Low Range Protein Ladder (2-40 kDa) (Thermo Scientific, Rockford, IL, U.S.A.) was loaded in parallel as a molecular weight marker. Electrophoresis was performed in Tris-tricine running buffer (0.1m Tris, 0.1M tricine, 0.1% SDS, pH 8.3) using a constant voltage of 125 V provided by Criterion Powerpac^TM^ basic power supply (Bio-Rad, Hercules, CA, U.S.A.). Upon electrophoresis, the gel was fixed by 5% glutalderhyde for 25 min, stained using 0.025% w/w of CBB G250 in 10% acetic acid for 20 min, and destained in 10% acetic acid until a light and homogenized background was achieved. The electrophoretic pattern of the peptides was captured by a Bio-Rad ChemiDoc imager, and the relative proportion of the peptide bands was analyzed densiometrically by Alphaview software (version 3.3.1, Cell Biosciences, Santa Clara, CA, U.S.A.).

Selected hydrolysate peptide mixture from each enzymatic hydrolysis was characterized by LC-MS/MS for peptide sequence identification and molecular weight distribution of the peptide products. The hydrolysate samples were diluted in aqueous 3% acetonitrile with 0.1% formic acid to 1:100 for those derived from pepsin (ZP24), alcalase (ZA12), and thermolysin (ZTh6), and 1:300 for those from chymotrypsin (ZCH4) and papain (ZPa24). Mass spectrometry analysis was performed on an Orbitrap Fusion (Thermo Scientific, Waltham, MA, U.S.A.) equipped with a Digital PicoView source (New Objective, Littleton, MA, U.S.A.) and coupled to a M-Class UPLC (Waters, Milford, MA, U.S.A.). Solvent composition of the two channels was 0.1% formic acid for channel A, and 0.1% formic acid and 99.9% acetonitrile for channel B. Each sample was loaded on a commercial MZ Symmetry C18 Trap Column (100Å, 5 µm, 180 µm x 20 mm, Waters) followed by nanoEase MZ C18 HSS T3 Column (100Å, 1.8 µm, 75 µm x 250 mm, Waters). The peptides were eluted at a flow rate of 300 nl/min. After an initial hold at 5% B for 3 min, a gradient from 5 to 25% B in 40 min and 36% B in 10 min was applied. The column was washed with 95% B for 5 min, after which the column was re-equilibrated to starting conditions for an additional 10 min. The acquired MS data were processed using Byonic 5.4 (Protein Matrics, Cupertino, CA, U.S.A.) and PEAKS Studio XPlus (Bioinformatic Solutions, Waterloo, Canada). The mass spectra were searched against a protein database consisting of α-zein P04700, O48966, β-zein P06673, and γ-zein P08031*.* The search took the oxidation modification on methionine (M) residues into account, and the specificity of the enzyme was set to none. The proteomic result was exported into Scaffold 5 (Proteome software, Portland, OR, U.S.A.) for visualization.

**5. Evaluation of fibrillization potential of zein peptide mixtures**

*5.1. Aggregation potential*

As a preliminary screening for the fibrillization potential of different zein peptide mixtures, the aggregation process of the different samples under heat treatment was monitored by the increase in turbidity and changes in hydrodynamic size. All peptide mixture samples (5% wt, MilliQ water, pH 8) were heated at 60 °C in an oil bath for 24 h. The absorbance of the samples (200 µL in a transparent 96-well plate) was measured at 500 nm using a Tecan plate reader (Infinite M200 pro, Tecan Group AG, Männedorf, Switzerland). The turbidity increase was calculated using absorbance prior to and after heat treatment. The measurement was performed in duplicate. The hydrodynamic size of the peptide mixtures (0.1-0.5 mg/ml) prior and after the heat treatment was evaluated using a Zetasizer Nano ZS (Malvern Instruments, Malvern, Worcestershire, UK). The size distribution was calculated by an average of 36 measurements.

*5.2. Physicochemical characterization*

The surface hydrophobicity of different zein peptide mixtures was evaluated by 8-Anilino-1-naphthalenesulfonic acid (ANS)-fluorescence assay. ANS solution at 1 mm was prepared using MilliQ water and filtered through a 0.22-micron syringe filter. The measurement was carried out in a black flat bottom 96-well plate using a Tecan microplate reader. Peptide sample with a concentration ranging from 0.2 to 2.5 mg/ml at pH 8 was mixed with ANS solution at a volume ratio of 4:1, followed by incubation at 25 °C with orbital agitation for 70 s. Immediately after, the fluorescence intensity was measured at an excitation/emission wavelength of 390/484 nm. The surface hydrophobicity of the peptide samples was normalized by the protein content as determined by Hatree-Lowry assay. The ζ-potential of these peptide samples at 0.1% w/w at pH 8 was measured using the Zetasizer Nano ZS. The measurement was carried out in triplicate.

**6. Zein nanofibril preparation and purification**

Zein peptide with the greatest aggregation potential was subsequently investigated for the ability to form zein nanofibrils. Zein peptide solution at 2-5% w/w was prepared in MilliQ water and adjusted to pH 8 using 5 m NaOH. Upon full dissolution, the peptide solution was filtered by a FilterBio Cellulose Acetate syringe filter with 0.22-micron pore size. The solution was incubated at 60 °C in an oil bath for 0-24 h under continuous stirring at 320 rpm. To investigate the effect of surfactants on such fibrillization, surfactants, including SDS, Span 80, Tween 20, and Tween 80, were also co-solubilized with zein peptides at a concentration of 2.5 to 8.5 mm prior to filtration. An aliquot of solution was taken during the incubation for the evaluation of fibril formation kinetics. A scale-up production (30~50 g) of nanofibril was performed similarly as described; sample after incubation for 6 h and 24 h was recovered, as the nanofibril at the early and late stages, respectively. The nanofibril solution was diluted to 10 mg/ml using pH 8 MilliQ water and purified using an ultrafiltration system, Amicon® Ultra Centrifugal Filter, with 100 kDa molecular weight cut-off. The unfibrillized zein peptide was separated from the nanofibril by centrifugation at 4000 $\times$g, 20 min at 20 °C; retentate containing nanofibrils was washed twice with pH 8 MilliQ water and recovered by centrifugation. The purified nanofibril was subjected to structural characterization, as discussed in section 2.9.

**7. Congo red binding assay**

Spectrophotometric analysis of zein nanofibril-Congo red complexes was conducted using a plate reader. A mixture of 5 µm Congo red and 0.1% (w/w) zein nanofibrils was incubated in phosphate-buffered saline (PBS, pH 7.4) containing 10% (v/v) ethanol for 10 minutes at room temperature. The absorbance spectrum was recorded over a wavelength range of 300 to 700 nm.

**8. Fibrillization kinetics by Thioflavin T fluorescence assay**

ThT shows special affinity to the β-sheet structure in the nanofibrils, resulting in significant attenuation in fluorescence signal. ^[^^[[1]](#footnote-1)]^ ThT stock solution of 0.54 mM was prepared in sodium phosphate buffer pH 7 with 150 mm of sodium chloride and filtered with a 0.45-micron syringe filter. Reaction mixture (200 µL, in MilliQ pH 8) containing fibrillization samples (0.05 % wt) and 54 µm of ThT stock solution were prepared in black flat bottom 96-well plate and incubated at 25 °C for 1 min under orbital agitation prior to fluorescence measurement. Fluorescence intensity upon binding with ThT was measured at ʎexc/ʎemi of 440/482 nm.

**9. Structural characterization**

*9.1. Morphological analysis*

The morphology of zein nanofibrils was analyzed by atomic force microscopy (AFM) and transmission electron microscopy (TEM). Fibrillization sample (20 µL, 1 wt %) was loaded to mica that was modified with 1 wt% of APTES ((3-Aminopropyl)triethoxysilane). The residual sample on mica was rinsed away using pH 8 MilliQ water, and the mica was dried under gentle flow of air. The AFM imaging was carried out using a Bruker MultiMode 8 scanning probe microscope (Bruker, Billerica, MA, U.S.A.), operating in tapping mode under ambient conditions with a commercial silicon nitride cantilever (Bruker, Billerica, MA, U.S.A.) at a vibration frequency of 150 kHz. AFM images were processed by NanoScope Analysis 1.5 and FiberApp for morphological analysis. ^[^^[[2]](#footnote-2)]^

For TEM analysis, fibrillization samples (4 µL, 0.5 to 1 wt %) was fixed onto negatively charged carbon-coated copper grids, followed by staining with 2 wt% uranyl acetate. The images were taken by bright field TEM (FEI, Morgagni 268, U.S.A.) operated at a voltage of 100 kV.

*9.2. Secondary structure*

The secondary structure of zein, zein peptide mixture, and crude and purified nanofibril were analyzed by Fourier Transfer Infrared (FTIR) and circular dichroism (CD) spectroscopy. Zein solution, at an equivalent weight percentage to zein peptide and nanofibril samples, was prepared in pH 8 MilliQ water containing 1% wt of SDS. FTIR spectra were collected using a Varian 640 FTIR spectrometer (Varian Medical Systems, Palo Alto, CA, U.S.A.) equipped with an ATR accessory (Golden Gate Diamond, Specac Ltd., Orpington, UK). Fibrillization sample (5 µL, 5 %wt) was transferred onto the ATR crystal and allowed to air-dry for 20 min. Upon full evaporation of the solvent, the measurement was carried out in absorption mode in the range of 600-4000 cm^-1^ with a resolution of 4 cm^-1^ and an average of 32 scans. Nanofibril samples were diluted to 0.05-0.2 mg/ml with pH 8 MilliQ water for CD measurements. The diluted sample was transferred to a quartz cuvette with 1 mm pathlength. Spectra were taken in the range of 190–260 nm using a Jasco J-815 CD spectrometer (Jasco, Tokyo, Japan). Measurements were conducted with a resolution of 0.5 nm and a bandwidth of 1 nm in triplicate.

*9.3. Amino acid sequence identification*

To reveal the fibrillization prone region of zein, the purified nanofibrils were subjected to chymotrypsin digestion and LC-MS/MS for sequence identification. The digestion was performed by mixing purified nanofibril (20 µL, in pH 8, MilliQ water) with digest buffer (25 µL, pH 8, 10 mm Tris, 2 mm CaCl_2_) and chymotrypsin (5 µL, 100 ng/µL) followed by incubation at 37 °C for 4 h. The digesta was dried and resuspended in aqueous 3% acetonitrile with 0.1% formic acid, and the peptide concentration was estimated with the Lunatic UV/Vis absorbance spectrometer (Unchained Labs, Pleasanton, CA, U.S.A.). Data acquisition and analysis were performed as described in section 2.4.

The detected peptide fragments matching with zein were displayed on zein molecule and sequences. The protein data bank structure of α-zein, accession number O48966 and γ-zein accession number P08031, were downloaded from UniProt. The molecular visualization was performed using VMD program, 1.9.4 version (Visual Molecular Dynamics, University of Illinois and Beckman Institute, Urbana, IL, U.S.A.), ^[^^[[3]](#footnote-3),^^[[4]](#footnote-4)]^ and the sequences were edited using DiscoveryStudio 2021 (BIOVIA, San Diego, CA, U.S.A.).

**10. Emulsifying property of zein nanofibrils**

Native zein, zein peptide, and zein nanofibrils were investigated for their emulsifying properties. A high internal phase emulsion system was prepared by zein samples at 1% wt with canola oil at a fraction of 1:3. The system was homogenized using an Ultra Turrax T25 basic homogenizer (IKA-Werke GmbH & CO. KG, Staufen, Breisgau, Germany) for 90 s. For the emulsifying activity index (EAI), the emulsion was diluted 200 times using 0.1% SDS, the turbidity was measured at 600 nm as A_0_, and the EAI was calculated using equation 1: ^[^^[[5]](#footnote-5)]^

$EAI \left( \frac{m^{2}}{g} \right)=\frac{2\times2.303\times A0\times DF}{c\times\varphi\times{10}^{4}}$ (Equation 1)

Where DF represents the dilution factor (200); c is the concentration of the emulsifier (10 mg/ml); and φ is the oil fraction (0.75).

The particle size of the emulsion droplet was observed using an optical microscopy (Axio Scope.A1, Carl Zeiss AG, Jena, Germany). 1 mL of emulsion was transferred into a 1.5 mL transparent glass vial to be observed visually for phase separation over 2 weeks to compare the stability among the emulsion samples.

**11. Gelling properties of zein nanofibrils**

Zein nanofibrils were tested for the ability to form fibrous hydrogel structures. The gelling condition at both pH 3 with 100 mm of CaCl_2_ and at pH 7.5 with 100 mm CaCl_2_ were investigated. Nanofibril samples (1 mL) were loaded into a cylindrical mold (diameter: 1 cm, total volume: 1.5 mL) and sealed using a dialysis membrane with MWCO of 6-8 kDa. The mold containing the nanofibrils sample was submerged in gelling buffer at the aforementioned conditions, and buffer exchange was allowed overnight. Hybrid nanofibrils were prepared by mixing 5 wt% of potato protein with F6H at volume ratio of 1:1, and incubating at pH 8 and 60°C for 1 h. A composite hydrogel from this hybrid nanofibrils was also prepared using the condition of pH 7.5 with 100 mm CaCl_2_. Dynamic oscillatory amplitude sweep of the hydrogels was carried out at 0.1 to 100 rad/s using an MCT Anton Paar rheometer, equipped with 25 mm diameter parallel plate geometry with a serrated surface. The gap was kept constant at 3 mm. Scanning electron microscopy (SEM) was used to investigate the microscopic morphology of the hydrogels. Prior to SEM imaging, selected hydrogel samples were subjected to subcritical carbon dioxide drying at 98% of ethanol as a solvent and coated with platinum.

1. 1. M. Biancalana, S. Koide, *Biochim. Biophys. Acta* **2010**, *1804,*1405. [↑](#footnote-ref-1)
2. 2. I. Usov, R. Mezzenga, *Macromolecules* **2015**, *48,*1269. [↑](#footnote-ref-2)
3. 3. W. Humphrey, A. Dalke, K. Schulten, *J. Mol. Graphics* **1996**, *14,*33.

   4. C. Lara, N. P. Reynolds, J. T. Berryman, A. Xu, A. Zhang, R. Mezzenga, *J. Am. Chem. Soc.* **2014**, *136,*4732.

   5. K. N. Pearce, J. E. Kinsella, *J. Agric. Food Chem.* **1978**, *26,*716. [↑](#footnote-ref-3)
4. [↑](#footnote-ref-4)
5. [↑](#footnote-ref-5)
